# Higher-Order DNA Secondary Structures and Their Transformations: The Hidden Complexities of Tetrad and Quadruplex DNA Structures, Complexes, and Modulatory Interactions Induced by Strand Invasion Events

**DOI:** 10.3390/biom14121532

**Published:** 2024-11-29

**Authors:** Jens Völker, Vera Gindikin, Kenneth J. Breslauer

**Affiliations:** 1 Department of Chemistry and Chemical Biology, Rutgers University, 123 Bevier Rd, Piscataway, NJ 08854, USA; jvolker@chem.rutgers.edu (J.V.); vgind@chem.rutgers.edu (V.G.); 2 The Rutgers Cancer Institute of New Jersey, New Brunswick, NJ 08901, USA

**Keywords:** DNA strand invasion, metastable DNA states, G-quadruplex/iDNA/duplex interconversions, rough energy landscapes, competing kinetic and thermodynamic states, coupled transformations of higher-order DNA states, DNA self-regulation pathways

## Abstract

We demonstrate that a short oligonucleotide complementary to a G-quadruplex domain can invade this iconic, noncanonical DNA secondary structure in ways that profoundly influence the properties and differential occupancies of the resulting DNA polymorphic products. Our spectroscopic mapping of the conformational space of the associated reactants and products, both before and after strand invasion, yield unanticipated outcomes which reveal several overarching features. First, strand invasion induces the disruption of DNA secondary structural elements in both the invading strand (which can assume an iDNA tetrad structure) and the invaded species (a G-quadruplex). The resultant cascade of coupled alterations represents a potential pathway for the controlled unfolding of kinetically trapped DNA states, a feature that may be characteristic of biological regulatory mechanisms. Furthermore, the addition of selectively designed, exogenous invading oligonucleotides can enable the manipulation of noncanonical DNA conformations for biomedical applications. Secondly, our results highlight the importance of metastability, including the interplay between slower and faster kinetic processes in determining preferentially populated DNA states. Collectively, our data reveal the importance of sample history in defining state populations, which, in turn, determine preferred pathways for further folding steps, irrespective of the position of the thermodynamic equilibrium. Finally, our spectroscopic data reveal the impact of topological constraints on the differential stabilities of base-paired domains. We discuss how our collective observations yield insights into the coupled and uncoupled cascade of strand-invasion-induced transformations between noncanonical DNA forms, potentially as components of molecular wiring diagrams that regulate biological processes.

## 1. Introduction

### 1.1. Background

Biological pathways often exhibit complex, so-called “rough”, energy landscapes, with multiple local minima and variable height transition state barriers between them [1,2,3,4,5]. The information content scales with such landscape complexity and often reflects the co-mingling and differential temporal phasing of thermodynamically controlled (stability) and kinetically controlled (speed/rate) cascades of interconverting states, which, in part, are dependent on the local cellular milieu (see, for example, the elegant analysis of transcription regulation by Greive and von Hippel [6]). In this context, sequence-dependent, higher-order, noncanonical DNA structures [7,8,9,10,11] may exhibit coupled interactions that can be modulated by strand invasion events, as has been proposed as part of homologous recombination, the gene regulation pathways, and for telomeric function [12,13,14,15,16,17,18,19]. The associated conformational “gymnastics” of higher-order DNA structures can produce unanticipated molecular “dance partners.” In this article, we posit that the mapping of the “coupled polymorphism” of noncanonical DNA states can yield insights into how higher-order DNA species participate in the “molecular tuning” of pathways associated with DNA (self-) regulation.

We initially chose to focus our attention on the conformational gymnastics [20] between the canonical and noncanonical DNA states associated with the G- and C-rich DNA segments found in the promoters of a number of critical oncogenes [21,22,23,24]. The justification for our system design came from the results of prior studies that collectively suggest that oncogene transcription is likely regulated, at least in part, by arrays of kinetically and thermodynamically controlled transformations between the Watson–Crick duplex, G-quadruplex, and iDNA states that can be adopted by these G- and C-rich DNA domains [25,26,27,28,29].

#### Nomenclature and Biological Justification for Studying Such Higher-Order DNA Structural Motifs: G- and C-Rich DNA Segments in Gene Control Regions Are Prone to Form Noncanonical DNA Secondary Structures in Competition with Duplex DNA

Guanine-rich nucleic acid sequences that contain four or five G tracts of three or more guanines in length, separated by a variable number of non-G bases, can be designated as [G_3-4_L_1-7_]_4-5_ (where L = A, T, or C). In some instances, L also can be a G residue. Such domains are statistically highly over-represented in the genome [30,31], as are the corresponding, complementary C-rich sequences in the opposing strand, which can be designated as [C_3-4_L_1-7_]_4-5_ (where L = A, T, or G, and, rarely, C). Such types of G-rich (and C-rich) domains frequently are found in the promoter and enhancer regions of many genes, while being especially prevalent in the promotor regions of critical oncogenes, where they likely contribute to the regulation of promoter activity [32,33,34,35]. G-rich oligonucleotides (and their corresponding C-rich complements) that are derived from such promoter sequences exhibit a propensity to adopt a host of related noncanonical G-quadruplex and iDNA structural motifs, with subtle variations in the sequence defining which of the energetically closely related G-quadruplex/iDNA conformers are preferred [36,37,38]. It is envisioned that in vivo these noncanonical structures compete with conventional B-DNA duplex formation via dynamic DNA conformational exchanges [21,39,40,41,42]. Such competing conformational interconversions lend credence to suggestions that regulatory outcomes of promoters containing such G/C-rich domains may, in part, be tuned by dynamic transitions between different DNA conformations.

Current models for how such dynamic conformational exchanges might regulate DNA transcription involve a broad range of proposals, including the following suggestions: once formed, G-quadruplexes function as passive barriers to RNA polymerase progression (a negative regulatory process) [43,44,45,46]; the secondary structure acts as a “transient” binding site for the critical transcription factors needed for RNA polymerase activity (a positive regulatory outcome) [47,48,49]; and G-quadruplexes facilitate large DNA loop formation, possibly via transcription factor binding, as a part of the enhancer element function (also a positive regulatory outcome) [50,51].

Conceptual models for the role of iDNA to date focus largely on its role as a CIS-acting regulator of DNA transcription through the dynamic competition between the structured iDNA and unstructured C-rich loop isomers, with protein or ligand binding interactions stabilizing the iDNA form, thereby resulting in increased transcription levels [52,53]. Evidence also is accumulating for mutually antagonistic interactions between the iDNA form in one DNA strand and G-quadruplex formation in the opposing DNA strand, supporting the perspectives noted above on the importance of the dynamic conformational exchanges between different DNA states for regulating DNA transcription [54,55,56,57].

Additional support for such proposals also comes from observations that ligands that selectively stabilize G-quadruplex and/or iDNA structures [58,59] alter promoter activity, with antibodies specific to G-quadruplex structures tending to bind preferentially to promoters that contain such G-rich domains [28,60,61,62]. The observation that ligands specific to G-quadruplex structures can modulate promoter activity has spurred a widespread search for G-quadruplex specific ligands [63,64,65,66,67,68,69,70]. This focus reflects a strategic effort to deliberately manipulate the dynamic equilibria that control these noncanonical forms, with the ultimate goal of altering regulatory outcomes for in vitro and in vivo biomedical applications.

In this regard, oligonucleotides or oligonucleotide analogs represent an intriguing group of potential sequence-specific DNA ligands. Here the foundational Peptide Nucleic Acid (PNA) studies of Nielson, Norden, Wilson, Armitage, and others are pertinent [71,72]. They demonstrate strand invasion by “designer” PNAs into duplex DNA [73,74], including strand invasion by PNAs targeting the C-rich strand of [G_3-4_L_1-7_]_4-5_·[C_3-4_L_1-7_] _4-5_ tracts in duplex DNA. Such strand-invasion-induced binding of the C-rich strand can force the extrusion/formation of G-quadruplexes in the opposing strand [75,76,77]. These experimental results elegantly demonstrate that a strand invasion event at one site can induce the formation of a noncanonical state at a distal site: features conceptually associated with regulatory switches.

By contrast, the study reported here focuses on a cascade of strand-invasion-induced events that can result in the disruption, rather than formation, of the G-quadruplex secondary structure. Specifically, we focus on intermolecular interactions between separate oligonucleotides that can form a specific G-quadruplex structure, embedded within a duplex domain without its C-rich complementary strand, and a C-rich oligonucleotide that is exclusively complementary, in the Watson and Crick sense, to the G-rich segment that folds into the quadruplex conformation.

Table 1a lists the sequences and the designated names of the three oligonucleotides we have selected for this study. Table 1b schematically illustrates the range of potential inter- and intramolecular interactions between these three DNA oligomers in their various unimolecular, bimolecular, and trimolecular states. The cMycG strand in Table 1a is named based on the near homology of the central G-rich domain with G tracts I-IV of the nuclease hypersensitivity element NHEIII of the *cMyc* oncogene promoter sequence. The cMycG single strand exists as an ensemble of closely related quadruplex conformers, of which, the parallel strand arrangement of the G 4 domain depicted in Table 1b is only one, albeit likely the dominant, isomer adopted at a low temperature in K^+^ salt [36,37,38]. The iDNA conformer of the invading strand (IS) also reflects low temperature ensembles of closely related conformations of the iDNA species formed by the C-rich domains, rather than implying that the cartoon structure reflects any single conformation. Henceforth, we designate the condition-dependent C-rich invading strand (IS) either as iIS, rcIS, or generically as IS to indicate that it refers to the invading strand predominately in either its intramolecular iDNA folded conformation, the unstructured, single-stranded, random coil (rc) state, or as simply IS, thereby acknowledging the co-mingling of these two extremes in a manner that depends on the solution conditions under consideration.

By design, as illustrated in Table 1b, the 22merCO single strand is largely unstructured (see text) and is intermolecularly complementary, in a Watson–Crick sense, to the 5′ and 3′ terminal domains of the cMycG sequence, with a centrally extruded G-rich quadruplex (see Table 1b) reflective of a bimolecular complex, in which the G-quadruplex is embedded within a duplex structure.

Table 1a: The oligonucleotide sequences listed in Table 1a are color coded to indicate domain regions important for our studies, with red corresponding to G tracts (cMycG) or their complementary C tracts (IS), light purple indicting putative loop domains in cMycG quadruplex and IS iDNA, and blue corresponding to the flexible hinge domain between the G tracts and the 5′ and 3′ arms of cMycG shown in black. The position of the fluorescent base 2-Aminopurine is shown in green. The light blue arrow above the 22merCO sequence indicates the two 11mer half’s of the 22merC0 that are Watson and Crick complementary (indicated in black) to the 3′ and 5′ arms of cMycG, respectively. Table 1b outlines, in a pictorial format, the different ways by which the three single-stranded DNA oligonucleotides can inter- and intramolecularly interact mono-, bi-, and/or trimolecularly, as indicated in the last column of the table. The far-left column of Table 1b illustrates examples of intramolecular structures that can be formed using each of these three single-stranded oligonucleotides in isolation, whereas the right side of Table 1b pictorially illustrates intermolecular structures formed by complexes between different combinations of these three strands. For the sake of clarity, we represent the noncanonical secondary structure domains (G-quadruplex and iDNA) as pseudo-3-dimensional ball–stick figures, whereas the Watson and Crick domains are depicted only as solid lines. The pictorial form of the single strands depicted on the left side of Table 1b reflect the low temperature ensembles of closely related G-quadruplex conformations, adopted by cMycG and iDNA conformations of the IS in the single-stranded form. As noted above, the cMycG strand is named based on the near homology of the central G-rich domain with G tracts I-IV of the nuclease hypersensitivity element NHEIII of the *cMyc* oncogene promoter sequence. Similarly, the iDNA designation also reflects low temperature ensembles of closely related conformations of iDNA species formed by the C-rich domains, rather than any single conformation being implied by the cartoon structure. By design, the 22merCO single strand is largely unstructured (see text) and is complementary, in a Watson–Crick sense, to the 5′ and 3′ terminal domains of the cMycG sequence, with a centrally extruded G-rich quadruplex. Going forward, we will allude to the schematic figures and the shorthand notations shown in this composite Table 1a,b, as we propose correlations between the presence of specific DNA species and the characteristic spectroscopic features we detect and report herein for their free and interacting states.

### 1.2. Rationale for Design of the System Studied

#### Spectroscopic Features of and Reasons for Studying the “Mutated”/Site-Altered cMyc Sequences

For this investigation, we selected a mutated cMyc sequence derived from G tracts I-IV of the nuclease hypersensitivity element NHEIII of the *cMyc* oncogene promoter [78]. We made this selection since prior work demonstrates this mutated sequence in K^+^ salt to adopt a unique and stable parallel G-quadruplex conformation [23]. The tendency of this mutated cMyc sequence to form such a singular G-quadruplex, without evidence for the presence of other competing G-quadruplex forms, has made it a preferred and widely studied model for biophysical studies of a “homogeneous” G-quadruplex [79,80,81,82,83,84,85,86]. Nevertheless, it should be noted that the presence of competing G-quadruplex polymorphs, frequently observed for natural G tracts derived from cMyc, as well as other oncogene promoters, may well be biologically relevant in that they provide alternative folding intermediates that may further modulate promoter activity.

As shown by the cMycG strand listed in Table 1a, in the mutated version of the *cMyc* promoter element, all four G tracts (henceforth collectively referred to as the G4 domain) are of equal length and are comprised of three Gs, with consecutive G tracts separated by either one adenine (that putatively forms loops 1 and 3 in the G-quadruplex) or by a TA base step (the putative loop 2). To differentiate local conformational changes within the G4 domain from global changes monitored by classical spectroscopic ensemble averaging methods (Circular Dichroism spectroscopy (CD), Ultraviolet Absorption spectroscopy (UV)), we selectively replaced the adenine in the TA base step (loop 2) with its fluorescent analog 2-Aminopurine (2Ap), which serves as a local spectroscopic probe [87,88,89,90,91,92]. The two short single-strand segments (11bp each), placed at 3′ and 5′ of the G4 domain, are not related to any *cMyc* promoter segments, but rather are able to form stable duplex arms with the complementary strand designated as 22merC0. The purpose of these duplex arms is to restrict the degrees of freedom of the G4 domain and to more closely mimic the constraints placed upon a putative G-quadruplex forming sequence within the context of genomic DNA within our system. These arms correspond to a “generic” duplex domain we previously have used in trinucleotide repeat DNA studies [93,94,95,96,97] and which we have demonstrated can accommodate looped-out domains without forming potentially competing secondary structures themselves. This choice of flanking sequences was based on the desire to avoid potential complications within our model system from additional uncontrolled folding propensities inherent in sequences flanking the G4/G5 domains in natural promoters. We also included two unpaired T residues, 3′ and 5′ of the G4 domain, to serve as flexible hinges, thereby minimizing any yet-unknown consequences of forming junctions between the G-quadruplex structure and the adjacent duplex domains. We posit that the presence of such flexible hinges makes it likely that the upstream/downstream duplex and the G4 domains act as (semi-) independent entities; thereby providing a test for the degree of coupling of the physical properties of the different sequence/structure domains. We refer to the resulting complex as cMycG·22merC0, in which the dot-centered period (·) indicates Watson–Crick base pair formation between the 22merC0 strand and the single-strand segments upstream and downstream of the G4 domain in cMycG.

The intermolecular invading strand (IS) is complementary, in a Watson–Crick sense, to the G4 domain, but lacks the bases complementary to either the flexible hinge domain or the upstream/downstream duplex domains. This IS oligomer can fold, under the appropriate conditions, into an intramolecular iDNA held together by pairs of hemi-protonated cytosine–cytosine base pairs, interdigitated to form the four-stranded complex [98,99,100,101,102,103]. When this oligomeric invading strand (IS) is bound to the G4 domain, we will refer to the resulting complex as the G4·C4 duplex, to underscore the Watson–Crick base-pairing interactions between the IS and the G4 domain of cMycG. As elaborated below, we are able to distinguish two forms of the G4·C4 duplex; namely, the linear duplex formed between cMycG-IS (as cartooned in column 2, row 1 of Table 1b) and the G4·C4 loop-duplex when the IS is bound to the cMycG·22merC0 complex (as cartooned in column 2, row 3 of Table 1b). We interpret our spectroscopic results as being consistent with both forms, formally being characterized by the same Watson–Crick base-pairing interactions, but differing in physical properties due to different topological constraints.

**Scheme 1:**
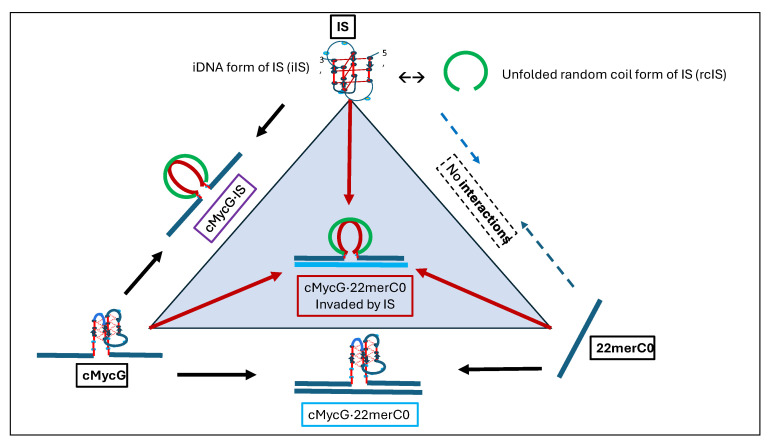
Illustrates the relationships between the three DNA oligonucleotides and their mono-, bi-, and/or trimolecular complexes.

The interactions between the three DNA oligomers discussed above and the relationship between the different constructs that can form between them can conveniently be represented in the form of a triangular, interconverting “state diagram,” as illustrated in Scheme 1 below. In this format, each triangular corner represents one of the three DNA oligonucleotide single strands by themselves. Binary complexes between any two oligonucleotides are shown along the sides of the triangle connecting the relevant corners, while the center of mass of the triangle reflects ternary interactions between all three oligonucleotides. Due to the lack of sequence complementarity between the oligonucleotide IS and 22merC0, no binary interactions between these two oligonucleotides are shown along the right side of the triangle.

These sequences, along with their associated designations and roles in the strand invasion process, are listed in Table 1 and in the Summary Section and the Glossary Section provided below.

### 1.3. Glossary of Designated States

**Scheme 2:**
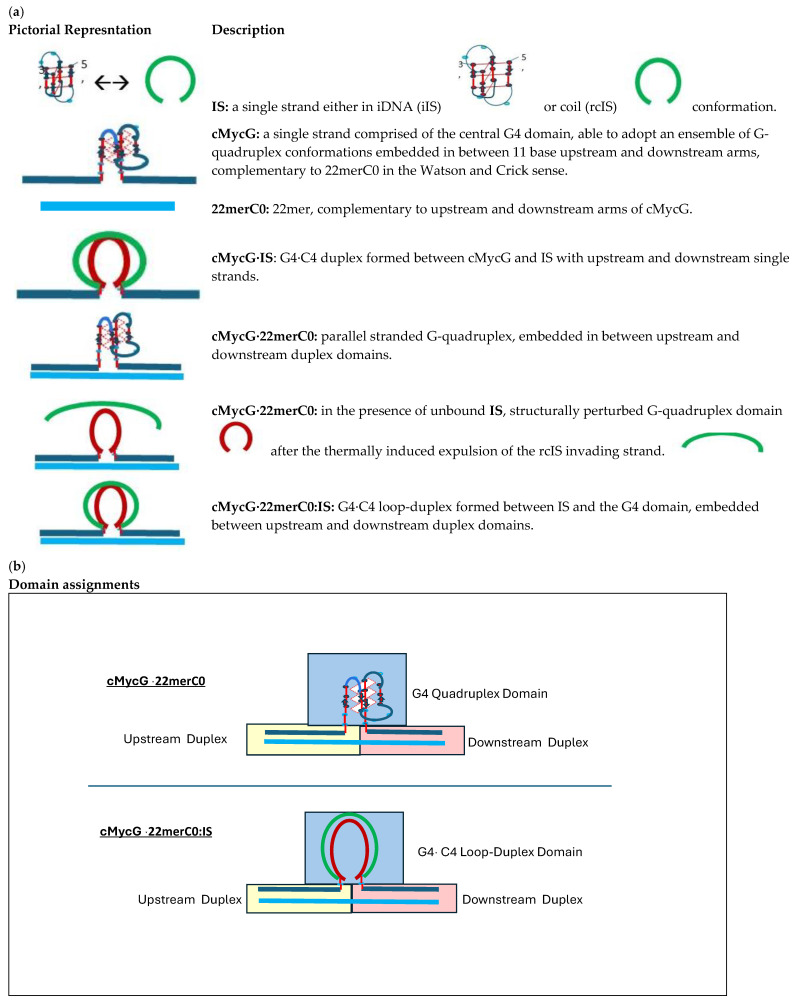
Pictorial guide of the DNA states and their graphical representations (**a**) and domain assignments (**b**).

Below we present a two-part Scheme 2 that highlights of the collective designations we use to specify each DNA species, as well as the complexes they can form under a given set of conditions, including the DNA domains we designate within these complexes. Going forward, this multicomponent glossary should facilitate the reader’s ability to correlate a given DNA structural species and its complexes and/or domains with the shorthand notation employed throughout this article.

In the sections that follow, we present local and global spectroscopic profiles of the isolated DNA states and their associated complexes that can form within mixtures of the various DNA species. Significantly, these profiles were measured as a function of temperature, time, sample preparation/history, solution conditions, as well as in the presence of competing species, including the impact of the order of addition. The resulting multidimensional array of measurements allows us to construct summary state diagrams/flowcharts, as introduced below. Collectively, these multivariable/multidimensional flowcharts pictorially present the overall cascade of inter-related transformations we propose based on our interpretation of the spectroscopic measurements we have conducted.

For clarity and continuity, at the beginning of each section below, we include a cartoon representation of the relevant transition under discussion. Following the presentation of our experimental observations, and towards the end of this article, we reintroduce these summary state diagrams/flowcharts as we present a global integration of our spectroscopic observations in support of our primary conclusions.

Particularly noteworthy, from a global perspective, is the impressive diversity of the multiple DNA states we spectroscopically detect that can result from the co-mingling of just three oligonucleotides. Throughout this paper, we underscore this recurring theme; namely, the range and complexity of coupled and uncoupled DNA states that can result from strand invasion events triggered by the co-mingling of a relatively few initial oligonucleotide species.

### 1.4. Summary Flowcharts

Chart 1: Flowchart 1 provides pictorial representations that summarize the temperature- and time-induced transformations we observe within our DNA system across four discretely designated temperature domains.

Chart 2: Flowchart 2 provides pictorial representations of strand invasion and the associated disruption of secondary structures.

Chart 3: Flowchart 3 pictorially presents the impact of metastability and sample history on the distribution of the DNA species formed.

Chart 4: Flowchart 4 pictorially illustrates the impact of topological constraints on proposed transformational outcomes.

In the sections that follow, we present the underlying experimental data that support the interpretations manifest in these summary charts.

## 2. Materials and Methods

### 2.1. Materials

The HPLC-purified oligonucleotides listed in Table 1a were obtained from IDT (Coralville, IA, USA) and dissolved in distilled water. Aliquots of individual oligonucleotides from stock concentrations of between 0.4 and 0.6mM were separately added to small volumes of the desired buffer. The resulting solution then was heat equilibrated prior to mixing different strands together at the desired incubation temperature. DNA concentrations were determined using the following molar extinction coefficients: ε260(IS) = 123,900 M^−1^ cm^−1^; ε260 (cMycG) = 399,200 M^−1^ cm^−1^. The extinction coefficient of 22merC0, ε260 (22merC0)) = 190,400 M^−1^ cm^−1^ was determined independently by a phosphate assay ([104,105]). Potential complexes between any two strands were formed by using separately established protocols that employed specified incubation temperatures and times. These same protocols also were used prior to the addition of a third strand in experiments in which we probed third stand interactions with pre-existing conformations. Unless otherwise specified, measurements were conducted in a pH 6.8 potassium buffer consisting of 10 mM Potassium Cacodylate, 0.1 mM EDTA, and sufficient KCL to give a final concentration of 50 mM in K^+^ cations. In cases where a different cation (e.g., Li^+^ or Na^+^) was required, the same pH 6.8 buffer was used, except that the Potassium Cacodylate and KCL were replaced by Lithium Cacodylate/LiCl or Sodium Cacodylate/NaCl, as appropriate. All of the buffer components were purchased from Sigma Aldrich, St. Louis, MO, USA in the highest purity grade available.

### 2.2. Spectroscopy

Circular Dichroism (CD), UV, and fluorescence excitation spectra are classical spectroscopic tools to interrogate the three-dimensional folding of biopolymers [106,107]. Here CD, UV, and fluorescence excitation spectra [at the 2AP emission maximum of 370 nm] were recorded as a function of temperature using an AVIV 435 fluorescence CD spectropolarimeter (Aviv Biomedical, Lakewood, NJ, USA), as previously described ([108]). The unique design of the instrument allows for the simultaneous detection, in the same sample, of CD spectra and UV spectra in one direction, as well as fluorescent spectra at right angles to the incident beam. Temperature-dependent spectra were recorded between 360 nm and 205 nm (monitoring 2Ap emission at 370 nm) for all 3 observables, which were collected every 1 °C for heating and 2 °C for cooling cycles in 1 nm increments using a 3 s averaging time, with a 60 s temperature equilibration time prior to the measurement. Equilibration only commenced after the temperature had reached the set value, to within a tolerance level of ±0.15 °C, and the data were collected after equilibration, only while this temperature tolerance around the set temperature was maintained. This experimental protocol resulted in an approximate heating rate of 1 °C/16 min for both the heating and cooling curves. Buffer spectra obtained in the same manner were subtracted from the sample spectra and the resulting wavelength/temperature/intensity matrix was subjected to singular value decomposition (SVD) to identify the minimum number of significant components needed to describe the data and to reduce experimental noise. The oligonucleotide strand concentrations were 1 µM in each strand in 1 × 1 cm fluorescence cells. This concentration was determined by the CD requirement for the sample to not exceed 1.5 optical density (OD) units or to be less than ~0.4 OD at the maximum absorbance to give reasonable results. Single wavelength heating and cooling curves at select wavelengths were extracted from the relevant wavelength/temperature/intensity matrix. Time-dependent measurements were collected using the same basic protocol, except for keeping the temperature constant at the incubation temperature and for measuring the spectra at predetermined time intervals.

## 3. Results and Discussion

### 3.1. PART I. Background of the Isolated DNA Component Species

#### 3.1.1. Spectroscopic Evidence for the Formation of a Parallel-Stranded G-Quadruplex by cMycG·22merC0

**Figure 1:**
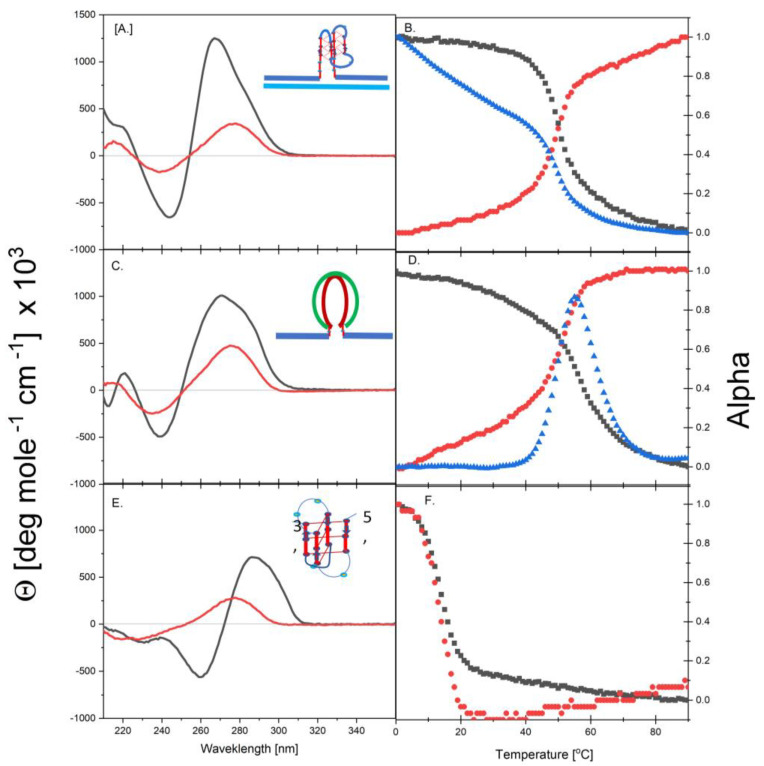
Figure 1 shows the CD spectra of the native (0 °C, black) and denatured state (90 °C, red) of the cMycG·22merC0 quadruplex (**A**), the duplex formed from cMycG·IS (**C**), and the IS single-strand iDNA complex (**E**). The corresponding normalized CD melting curves, recorded at the wavelength of the respective CD maximum (black curves), the absorbance melting curves at 270 nm (red curves), and the fluorescence melting curves (blue curves) measured for these 3 constructs, with each shown in (**B**,**D**,**F**). Note the unusual shape of the fluorescence melting curve in (**D**), where the melting of the G4·C4 duplex initially results in an increase in 2Ap fluorescence, followed by significant quenching at a higher temperature. The initial increase in 2Ap fluorescence is what is expected of melting of a duplex containing a stacked 2Ap·T base pair, while the rapid subsequent quenching of 2Ap fluorescence likely is due to the known rapid dark state quenching of 2Ap by neighboring guanines when both bases are freely mobile. As expected, (**E**) does not show a fluorescent melting curve, as the free IS does not contain a fluorescent 2Ap base.

**Scheme 3:**
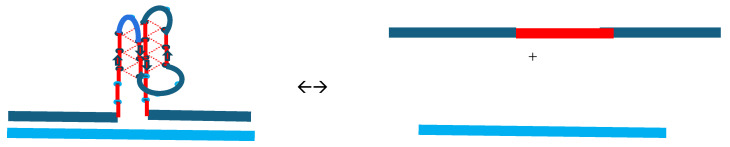
cMycG·22merC0.

Figure 1A shows the typical CD spectra we measured at 0 °C (native conditions) and 90 °C (denatured conditions) for cMycG, mixed 1:1 with the 22merC0 in 50mM K^+^ buffer. The native CD spectrum, with its strong positive ellipticity at 264 nm, is considered reflective of a parallel-stranded G-quadruplex [109,110,111,112]. In fact, the CD spectrum of the associated complex, cMycG·22merC0, can be represented, almost perfectly, as the sum of the CD spectrum of the 22mer duplex (22merC0·22merG0) and the CD spectrum of the isolated G4 domain that make up its component parts. The isolated G4 domain in K^+^ salt is known to adopt a parallel-stranded G-quadruplex conformation ([23,79,113,114,115]). The very strong enhancement in 2Ap fluorescence we observed (roughly 15 times more intense in K^+^ relative to Li^+^ or Na^+^;) also is consistent with the G4 domain in cMycG·22merC0 adopting the parallel G-quadruplex conformation under native conditions. Further shown in Figure 1B are the (normalized) corresponding CD-melting curve at 264 nm (black curve), the UV-melting curve at 270 nm (red curve), and the fluorescence melting curve (excitation at 308 nm, emission at 370 nm; blue curve) for cMycG·22merC0. All three curves reveal a single cooperative melting transition with identical Tm values of 48 °C. We propose that this transition, detected via multiple observables, corresponds to the coupled unfolding of the parallel G4 quadruplex domain, together with the upstream and downstream duplex arms of cMycG·22merC0, Scheme 3.

#### 3.1.2. Spectroscopic Evidence for the Formation of a Duplex with Overhanging Single-Stranded Ends Between cMycG and the IS

**Scheme 4:**
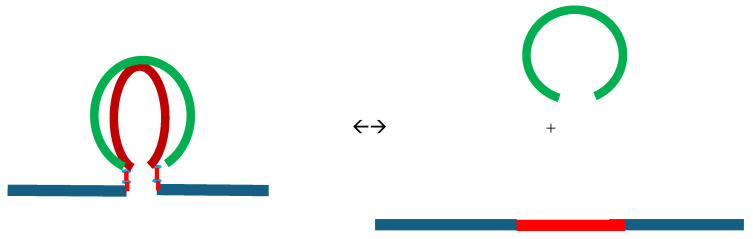
cMycG·IS.

Figure 1C shows typical CD spectra at 0 °C (native conditions) and 90 °C (denatured conditions) for a 1:1 mixture of cMycG and the IS. The corresponding CD, UV, and fluorescence melting curves are shown in Figure 1D. The 1:1 mixture of cMycG and the IS exhibits a single cooperative melting transition with a Tm of 53 °C. Note that this Tm is higher than that which we observed for cMycG·22merC0. The native CD spectrum of cMycG·IS (Figure 1C) differs from that of cMycG·22merC0 by an overall decrease in intensity and a significant shift in the maximum to 272nm. Although the measured CD spectrum shows evidence for the presence of a small amount of G4 quadruplex and iDNA due to residual single-stranded cMycG and IS folding, the overall spectrum of the cMycG·IS sample closely resembles that of a GC-rich duplex [111,115]. We posit that the dominant spectral component represents the spectrum of a central G4·C4 duplex domain between cMycG and the IS, which is surrounded by the unpaired upstream and downstream single-strand domains that are part of the cMycG oligonucleotide. The presence of a small number of G-quadruplex and iDNA states at equilibrium in oligonucleotides with the relevant degrees of freedom is due to the relative differential energetics of the duplex, G-quadruplex, and iDNA states, as previously shown by Chalikian and coworkers [115,116] and independently confirmed by us, Scheme 4.

#### 3.1.3. Spectroscopic Evidence for the Formation of an Unstable iDNA by the IS

**Scheme 5:**
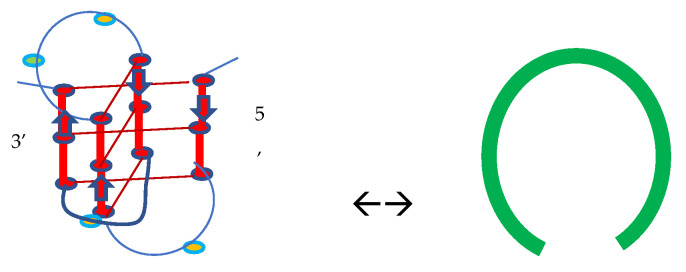
IS.

Figure 1E shows the typical CD spectra at 0 °C (native conditions) and 90 °C (denatured conditions) measured for the IS by itself. The corresponding CD and UV melting curves are shown in Figure 1F. Since the IS lacks a 2-Aminopurine base, no fluorescence melting curves are shown in Figure 1F. The CD spectra of the IS at 0 °C exhibit features typical of an iDNA conformation, with a CD maximum at 288 nm and a minimum at 260 nm [111,112,117,118,119], while also exhibiting a low melting transition with a Tm of 15 °C under the conditions employed here; namely, 50 mM K^+^ salt, pH 6.8. Reducing the pH of the solution shifts this lower melting transition to higher temperatures but does not alter the shape of the CD spectrum observed at 0 °C. Based on these collective observations, we propose that the isolated IS strand adopts a relatively unstable iDNA conformation at low temperatures under our experimental conditions, Scheme 5.

#### 3.1.4. cMycG·22merC0, cMycG·IS, and the IS Exhibit Unique Spectroscopic Properties

In the aggregate, our observations are consistent with the G4 domain in cMycG·22merC0 folding into the predicted stable parallel G-quadruplex, with the invading IS strand in isolation adopting an unstable iDNA structure at low temperatures. Above its low melting temperature, the isolated IS strand remains largely unstructured. Significantly, we conclude that the IS can form a Watson–Crick duplex with the bases in the G4 domain of the cMycG strand, and, therefore, is able to bind to the looped-out (and structured) G4 domain in cMycG·22merC0, as illustrated in Chart 1 and Chart 2.

In the sections that follow, we probe to what extent the IS is able to invade and disrupt the G-quadruplex form of cMycG·22merC0 by forming a competing G4·C4 loop-duplex. Our just-described observations reveal that the native state CD spectra of cMycG·22merC0 (Figure 1A), of the cMycG·IS complex (Figure 1C), and of the IS (Figure 1E) exhibit distinct differences from one another. These different optical signals enable us to spectroscopically identify the dominant species in solutions that contain all three strands: namely, cMycG, 22merC0, and the IS. In particular, the changes we detected in the 2Ap fluorescence with time, temperature, and sample preparation enable us to assign conformational changes associated specifically with the G4 domain.

### 3.2. Part II: Spectroscopic Evidence for Strand Invasion of the G-Quadruplex by the IS

**Figure 2:**
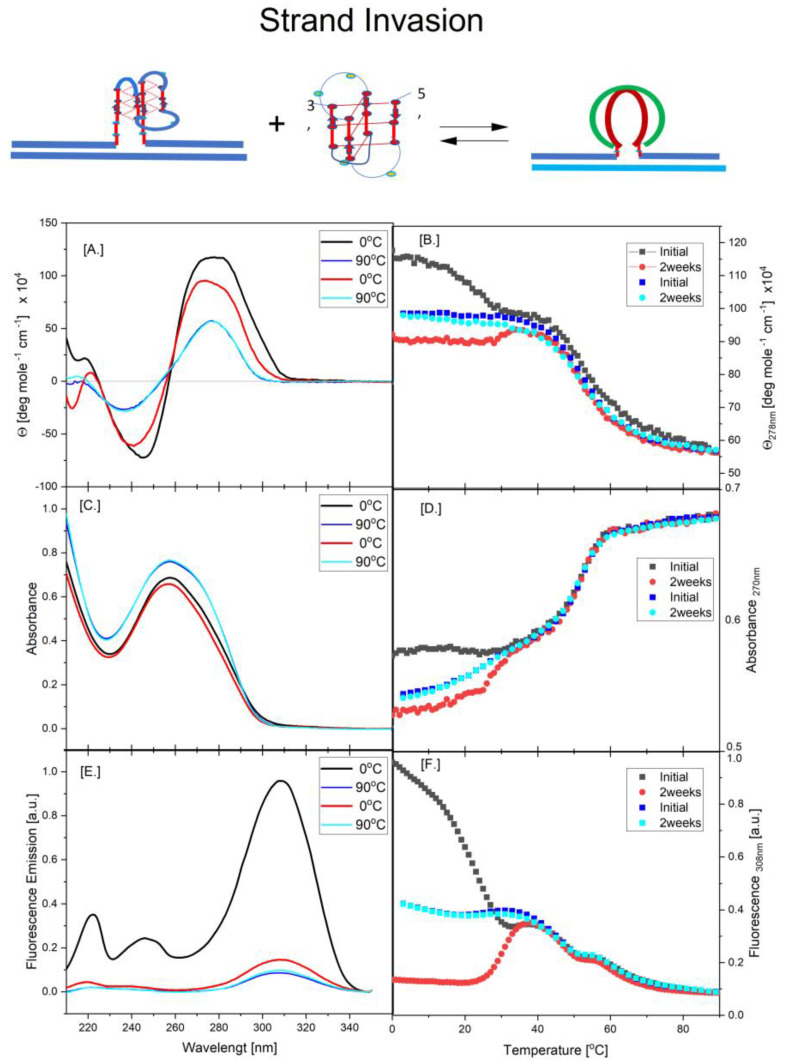
The native (0 °C) CD spectra (**A**), UV spectra (**C**), and fluorescence excitation spectra (**E**) of a freshly prepared cMycG·22merC0:IS sample (black curves), as well as after incubation at 4 °C for 2 weeks (red curve). Also shown in (**A**,**C**,**E**) are the spectra of both samples under denaturing conditions at 90 °C (dark and light blue). The corresponding CD, UV, and fluorescence melting (black and red) and reannealing (light and dark blue) curves at characteristic wavelengths are shown in (**B**,**D**,**F**), respectively.

**Scheme 6:**
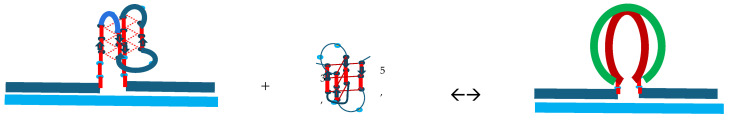
cMycG·22merC0 plus IS.

The addition of the C-rich oligonucleotide, the IS, to the preformed, parallel-stranded, G-quadruplex formed by cMycG·22merC0 results in the characteristic changes in spectra and melting behavior shown in Figure 2. Specifically, Figure 2A,C,E, respectively, reveal characteristic changes in the CD, UV, and fluorescence spectra, immediately after the addition of the IS and after a 2-week incubation period at 4 °C of a freshly prepared sample. After a 2-week incubation period, no significant further spectral changes were observed. Also shown in Figure 2B,D, and F are the corresponding heating and cooling curves, at characteristic wavelengths, for each of the three spectroscopic observables. The CD, UV, and fluorescence spectra were recorded simultaneously on the same sample using an AVIV fluorescence CD instrument, while melting curves at select wavelengths were extracted from collections of spectra recorded in 1 °C intervals for those samples, Scheme 6.

The following general observations are pertinent:

1. All three spectral observables obtained immediately after mixing (black curves) differ from the spectral observables after incubation for 2 weeks at 4 °C (red curves), with the most profound changes observed for the local 2Ap fluorescence probe inserted into the center of the G4 domain. By contrast, the 90 °C denatured state spectra (dark blue and cyan) are indistinguishable, as one would expect for the fully denatured forms. These collective observations are consistent with slow interactions between cMycG·22merC0 and the IS at the low incubation temperatures employed here. These conclusions are pictorially reflected in the corresponding flowcharts.
2. The associated melting curves reveal multiple transitions, with only the low temperature transition(s) depending on incubation times. In addition, the curves reveal hysteresis at low temperatures upon heating and cooling. By contrast, the higher temperature transitions are identical for the freshly prepared samples and for those incubated at 4 °C for 2 weeks prior to the melting experiments and are completely reversible, as reflected by the identical heating and cooling curves. On the other hand, for all the observables, the features of the initial low temperature transition depend strongly on the history of the sample, consistent with the pictorial representations within the relevant flow charts.
3. While all the optical observables reveal multiple temperature induced transitions, the 2Ap fluorescence melting curves exhibit the greatest resolution of identifiable transitions. We posit that this greater resolution, at least in part, results from the additional quenching of 2Ap by freely mobile guanines surrounding the 2Ap site following the disruption of base-paired/secondary structure elements involving the guanines that surround the local 2Ap residue [120,121,122,123,124] The implications of these results are further discussed in later sections.
4. The cooling/reannealing curves identically “reverse” the heating/melting transitions observed at high temperatures, yet they diverge for the low temperature transition in a reproducible manner. Specifically, the reannealing curves fall in between the heating curves initially observed upon melting and those observed after preincubation at 4 °C, while also exhibiting an intriguing and reproducible “wiggle” in the fluorescence annealing curves. Starting at the temperature where the heating and cooling curves begin to diverge during cooling, the 2Ap signal initially and gradually decreases to a temperature of about 20 °C, whereas at lower temperatures the 2Ap fluorescence begins to increase again. The significance of this behavior is discussed below, while also being pictorially illustrated in the relevant flow charts.
5. Particularly noteworthy, as previously underscored, and worth repeating for emphasis, is the impressive diversity of the multiple DNA states we spectroscopically detected that can result from the co-mingling of just three oligonucleotides. We re-emphasize how the kinetically and thermodynamically controlled array of interacting species and their product complexes are dependent on temperature, time, sequence, and sample history/preparation, including incubation of individual species prior to mixing. We regularly allude to this recurring theme: namely, the range and complexity of coupled and uncoupled DNA states that can result from strand invasion events triggered by the co-mingling of a relatively few initial oligonucleotide species.

In the sections that follow, we focus our attention on the analysis of the fluorescence melting curve (Figure 2F), as it is the most informative for understanding and assigning the complexity of the different transitions associated within this apparently “simple” DNA system.

### 3.3. Part III: Towards a Globally Integrated Understanding of the Collective Observations

#### 3.3.1. 2AP Fluorescence-Detected, Thermally Induced Alterations in the G-Quadruplex Strand of the cMyG·22merC0 Plus IS Complex

**Figure 3:**
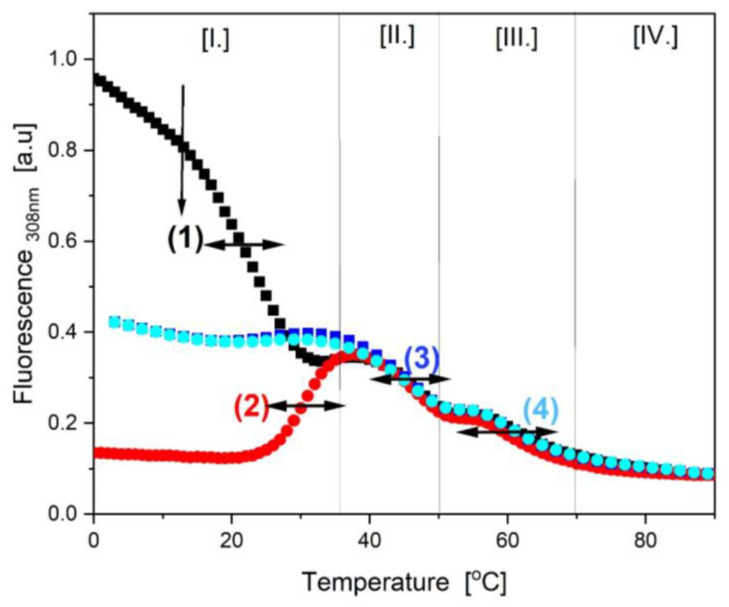
An expanded version of Figure 2F. As we show below, the conformational transitions designated 1 and 2 in the temperature domain [I.] are related to one another by the time variable and are discussed together in the text. Vertical lines indicate boundaries between different temperature domains that are defined by clearly distinguishable conformational transformations and are designated by Roman numerals I through IV. Conformational transitions are indicated by Arabic numerals 1 through 4.

To aid in resolving and assigning the nature of the conformational transitions observed when cMycG·22merC0 is mixed with the IS and incubated for variable times, we direct the reader’s attention to Figure 3. This figure represents an expanded version of Figure 2F. Note that in Figure 3 we indicate clearly identifiable conformational transitions by arrows associated with Arabic numerals. We also define the temperature domains relevant to these transitions, which are designated by Roman numerals. Below we discuss features of the transitions associated with each temperature domain. The conformational processes we propose for each temperature domain are schematically represented in the associated tables and identified by the relevant Arabic numerals in the main text.

The black curve in Figure 3 reflects the melting behavior immediately after mixing, while the red curve reflects melting after a 2-week incubation period at 4 °C. These behaviors collectively reflect the extremes of the incubation time effect we tested. The heating curves for samples incubated at 4 °C for intermediate time periods result in melting curves that fall between these two sets of curves. This intriguing incubation time dependence is important for understanding the set of reannealing curves shown in light and dark blue in Figure 3. In essence, the results shown in Figure 3 reflect a projection of the 3rd dimension “incubation time” axis onto the 2-dimensional fluorescence/temperature axis of a “classic” melting curve.

The inspection of Figure 3 reveals four distinct temperature domains, indicated by the vertical lines in Figure 3 and designated by Roman numerals I–IV, defined by clearly separated spectral transitions in the fluorescence signal, as indicated by Arabic numeral/arrows. Upon inspection of the data depicted in Figure 2, one observes similar transitions, albeit less well resolved, in both the UV and CD signals at characteristic wavelengths. For the UV and CD melting curves we find that different wavelengths are differentially sensitive to different conformational transitions. This reality results in the apparent absence of a particular transition or a lack of resolution at one wavelength, with that same transition being clearly detectable at a different wavelength, where another transition detected at the first wavelength shows no clear signal.

Recall that we incorporated the 2-Aminopurine base (2Ap) in the center of the G4 domain of cMycG with the specific goal of monitoring local conformational rearrangements of the G4 quadruplex domain embedded within neighboring duplex arms. Changes in UV or CD profiles reflect contributions not only from the G-quadruplex domain but also from the neighboring duplex arms, as well as those arising from the added IS invading strand. This multiplicity of contributions may partially mask the behavior of the G4 domain, which we primarily are interested in probing. Despite this complexity, we nevertheless still observe similar, albeit less well resolved, transitions in the CD and UV melting curves to those shown in Figure 3; an observation consistent with the observed fluorescence changes corresponding to conformational transitions of the DNA construct, or at least parts of it, rather than just the local rearrangements of the 2Ap base within the otherwise unperturbed G4 domain.

In the sections that follow, we discuss individually the DNA states associated with the different conformational transitions observed in Figure 3. To assist in this endeavor, the reader is re-introduced to Chart 1, which, using the same layout as the fluorescence melting curves shown in Figure 3, depicts the various transitions between different DNA states we have identified and designated in pictorial form. Specifically, we will show that the observed spectral changes in temperature domain [I.] are consistent with the time-constrained invasion of the G-quadruplex domain by the IS (transitions 1) and the subsequent melting of the resulting G4·C4 loop-duplex (transitions 2). We note that the G4·C4 loop-duplex embedded in between the upstream and downstream duplex arms exhibits features characteristic of D-loops (displacement loops) that are proposed to play important roles in telomeric function ([125,126]), as well as having a role in homologous/nonhomologous strand exchange reactions ([12,13,14]), two additional biological processes that also involve strand invasion events.

We further present spectroscopic evidence suggesting that temperature domain [II.] corresponds to formation and subsequent melting of the cMycG·22merC0 complex *in the presence of the expelled single-stranded IS* (transition 3), while temperature domain [III.] corresponds to the formation and subsequent melting of the cMycG·IS complex (i.e., the G4·C4 duplex) *in the presence of the 22merC0 single strand* (transition 4). In the temperature domain [IV.], all DNA conformations are fully denatured. See the flowchart differences as a function of the temperature domains. As we discuss below, the above *italicized* emphasis on the wording “in the presence of single strand...” is of significance, as the relevant complexes exhibit different properties when these single strands are absent, reminiscent of the so-called memory effects observed in some solvolysis reactions and their associated solvent separated and intimate ion pairs [127,128].

To aid in making the assignments noted above, we have made use of the melting curves of the isolated components of the cMycG·22mer:IS mixture (e.g., cMycG·22mer, cMyc·IS, and IS). We also made use of experiments orthogonal to classic melting curves in terms of monitoring the effects of time after the mixing of specific combinations of strands and/or by varying the order by which the strands initially are mixed and allowed to incubate at given temperatures. Unlike traditional melting experiments, where the heating rate is generally much slower than the kinetics of the melting process [129,130,131,132,133], we find that for some of the observed conformational changes the kinetics of the transition is much slower than the relatively slow heating rate (~1 °C/16 min) employed here.

Further, we observe that the order by which partial intermediates form can be critical for the population of states initially adopted and the time required to eventually form the equilibrium distribution. The preferential population of some states, to the temporary exclusion of others, becomes important for understanding the reannealing curves. For such curves, the order by which the complexes form is determined by a balance of the thermal and thermodynamic preferences of individual subsets of the components as the temperature is lowered, as well as their relative rates of formation. Experimentally, these influences can be tested by varying the order with which the three component strands are added together at different temperatures and by varying the incubation time they are allowed to interact. The importance of the history of the sample in defining the preferred distribution of conformational states also suggests that the use of conventional heat annealing processes routinely applied to oligonucleotide systems can lead to populations of non-equilibrium “initial” states and possibly erroneous conclusions about the samples, particularly if unrecognized slow kinetic processes contribute to the overall system. These realities are reflected in the corresponding flowcharts.

#### 3.3.2. Temperature Domain [I.]-Coupled Strand Invasion and the Expulsion of the Invading IS Strand

**Scheme 7:**
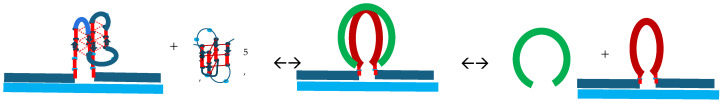
cMycG·22merC0 plus the IS. The invasion and expulsion of the IS.

- Comparison of “cMycG·22merC0:IS” with “IS”: evidence for strand invasion by the IS, Scheme 7.

**Figure 4:**
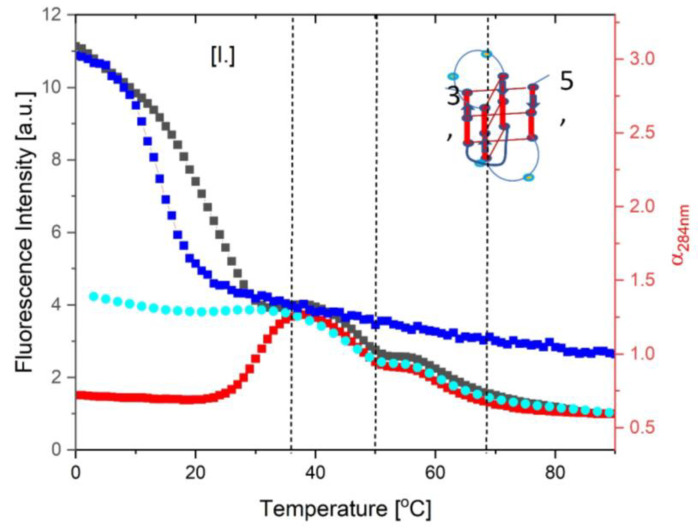
The overlay of the CD melting curve at 288 nm, observed for the isolated IS (blue curve, right Y axis) over the fluorescence melting (black and red, left Y axis) and reannealing curves (cyan, left Y axis) of cMycG·22merC0 with the IS added. The black curve corresponds to the melting of the cMycG·22merC0:IS sample immediately after mixing, whereas the red curve is the melting profile obtained after 2 weeks of incubation at 4 °C. The fluorescence melting curves of cMycG·22merC0 with the IS added in Figure 4 are identical with those depicted in Figure 3.

Figure 4 overlays the CD melting curve at 288 nm of the isolated (pre-equilibrated) IS strand (blue) over the two sets of cMycG·22merC0 plus the IS fluorescence melting/annealing curves recorded immediately after mixing (black curve) and after two weeks of incubation at 4 °C (red curve). The annealing curve is shown in cyan. The set of fluorescence melting curves in Figure 4 are the same as those shown in Figure 3.

**Figure 6:**
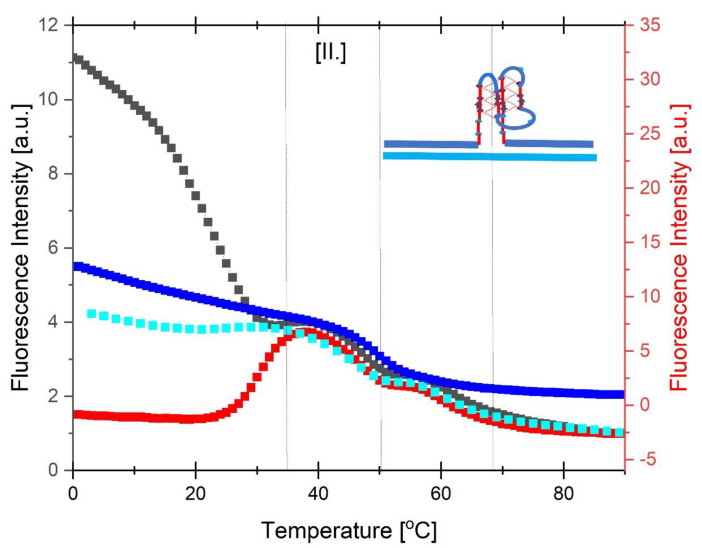
An overlay of appropriately scaled fluorescence melting curves for isolated cMycG·22merC0 (blue curve, right Y axis) over the fluorescence melting (black and red, left Y axis) and reannealing curves (cyan, left Y axis) of cMycG·22merC0 with the IS added. The black curve corresponds to the melting of the cMycG·22merC0:IS sample immediately after mixing, whereas the red curve is the melting curve obtained after 2 weeks of incubation at 4 °C. The fluorescence melting curves of cMycG·22merC0 with the IS, added in Figure 6, are identical to those depicted in Figure 3.

**Figure 7:**
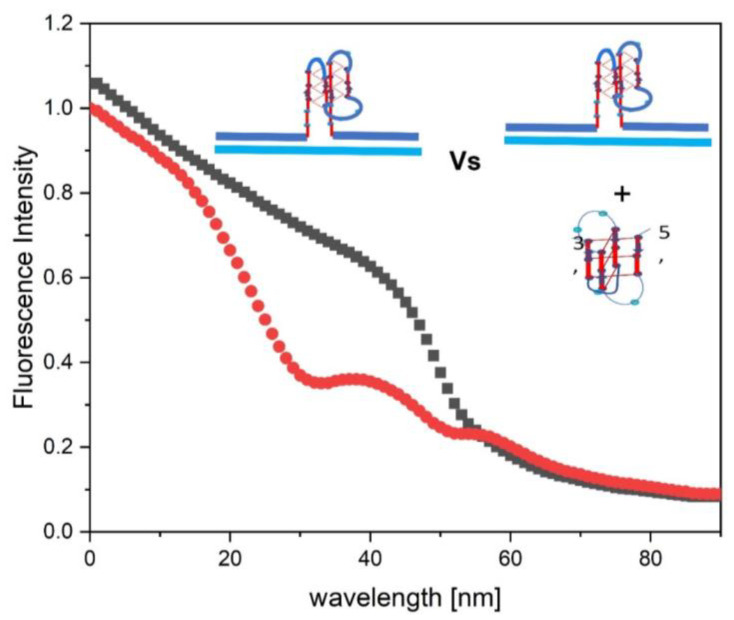
Compares, in absolute fluorescence terms, the fluorescent melting curves of the cMycG·22merC0 complex, which never has been exposed to the IS (black dots), to that measured for cMycG·22merC0:IS immediately after mixing (red dots). The small difference in initial fluorescence intensity at 0 °C reflects an inner filter effect, not exceeding 6%, caused by the absorbance of the native IS. This inner filter effect is likely absent at higher temperatures when the IS is denatured, as neither the denatured IS nor cMycG·IS (or native cMycG·22merC0:IS) significantly absorb at 308 nm.

Note that the decrease in 2Ap fluorescence seen in the cMycG·22merC0 plus the IS sample, measured immediately after mixing (black curve, corresponding to transition 1 in Figure 3), is similar in shape to the melting of the isolated IS iDNA complex detected by CD, although it is offset in temperature (and, therefore, also in time) to higher temperatures. Only the cMycG strand contains a 2Ap fluorescent base in the center of the G4 domain, so the observed fluorescence change reflects alterations of the G4 domain in the cMycG·22merC0 complex, not changes in the IS. In contrast to the more familiar fluorescent melting behavior of 2Ap in duplex DNA where melting results in an increase in fluorescence [87,88,120], when the 2Ap site is part of a parallel G 4 quadruplex, denaturation of the secondary structure in this system or duplex formation is associated with a decrease in 2Ap fluorescence. However, isolated cMycG·22merC0 that has never been exposed to the IS does not exhibit either a CD, a UV, or a fluorescence transition in the temperature domain [I.] (see also Figure 6 and Figure 7). We therefore propose that the observed correlation between the IS melting transition detected by CD and the temperature/time offset of the 2Ap fluorescence change in cMycG·22merC0 reflects the IS-induced slow (relative to the heating rate) unfolding of the G4 quadruplex into cMycG to form a G4·C4 loop-duplex within the loop domain of the cMycG·22merC0 construct.

Incubation of cMycG·22merC0 with the IS at 4 °C results in an even more pronounced decrease in the initial 2Ap fluorescence intensity without heating compared to the temperature-induced maximum decrease in fluorescence intensity seen for the sample heated immediately after mixing. This observation is reflective of additional 2Ap quenching due to the more complete G4·C4 loop base pair formation when the sample is incubated at 4 °C for 2 weeks, without changes in temperature (the red curve). Collectively, these observations suggest that G4·C4 loop-duplex formation remains incomplete during the heating of the freshly mixed sample (transition 1 in Figure 3, black curve). The melting of the sample incubated at 4 °C for 2 weeks, on the other hand, leads to a temperature-induced increase in fluorescence that cannot be duplicated by any combination of isolated components of this system (transition 2 in Figure 3).

We propose that the increase in 2AP fluorescence seen for the 2-week-incubated sample reflects the disruption of the fully formed G4·C4 loop-duplex domain and the expulsion of the invaded IS strand. We confirmed that the low temperature transition seen after 2 weeks of incubation (red curve) indeed reflects the expulsion of the invaded IS strand by observing the rapid displacement of the IS from a preformed cMycG·IS complex upon the addition of free 22merC0 at 36 °C, which is above the temperature of the first transition, seen in Figure 3. Interestingly, the addition of 22merC0 to the preformed cMycG·IS duplex at 4 °C (rather than 36 °C) leads to the fairly rapid formation of the species that is formed only after 2 weeks of incubation, when the order of addition is reversed and the IS is added to preformed cMycG·22merC0 at 4 °C. The formation of the cMycG·IS duplex, however, is slow at both temperatures (4 °C and 36 °C), indicating that the slow kinetics of G4·C4 duplex formation is, at least partially, due to the secondary structures adopted by the G4 domain. The flowcharts illustrate many of these spectroscopically based assignments.

As shown in Figure 4, the temperature-induced initial increase in fluorescence for the 2-week-incubated sample occurs in roughly the same temperature range and results in the same final fluorescence intensity at 36 °C as the decrease in fluorescence detected upon melting immediately after mixing with the IS. The samples that were incubated for intermediate times, or the samples recovered after reannealing, fall in between these two extreme melting behaviors. In other words, at the temperature boundary between domains 1 and 2, differences in the nature of the samples that depend on the initial incubation time and are reflected in different initial fluorescence signals no longer exist. The observed heating/cooling/incubation time properties described here also are reproduced in the samples only heated past the initial melting transition to 36 °C and are, therefore, independent of the conformational transitions observed at higher temperatures.

- The formation of the G4·C4 loop-duplex by strand invasion is kinetically inhibited but thermodynamically favored.

**Figure 5:**
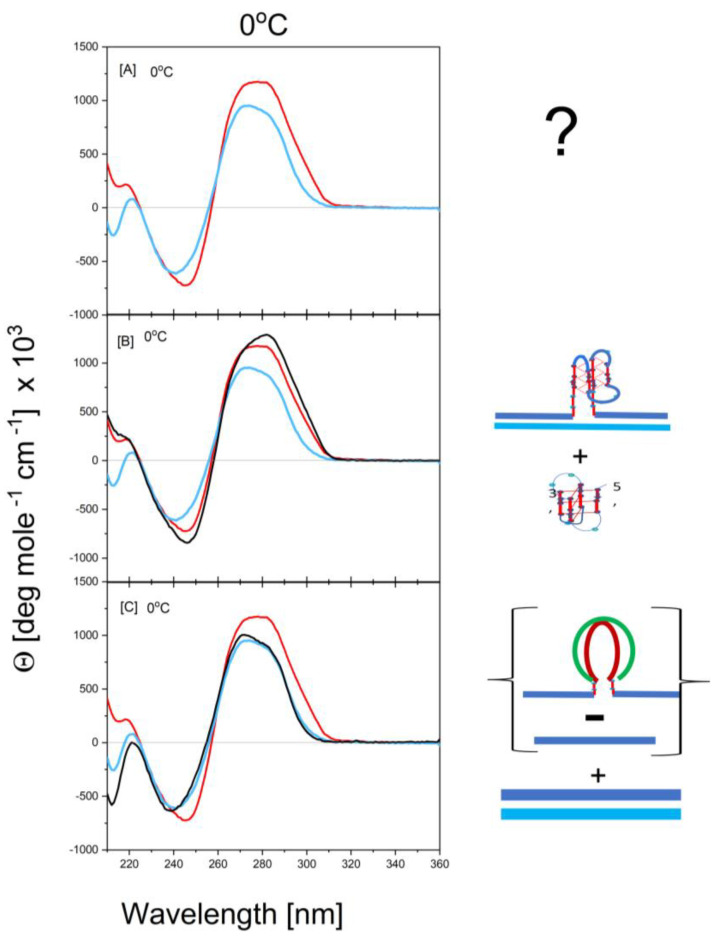
(**A**) shows the differences in the CD spectrum for cMycG·22merC0:IS at 0 °C obtained immediately after mixing (red curve) and after a 2-week incubation at 4 °C (blue curve). As shown in (**B**), the CD spectrum of cMycG·22merC0:IS at 0 °C immediately after mixing (red curve) is best approximated by the sum of the spectra of isolated cMycG·22merC0 and the IS (black curve), whereas (**C**) shows that the CD spectrum of cMycG·22merC0:IS after 2 weeks of incubation at 4 °C (blue) is best approximated by the sum of the CD spectra of the cMycG·i CShort duplex plus the 22merC0·22merG0 duplex minus the 22merG0 single strand (black curve). The subtraction of the 22merG0 single strand is needed to account for the single-stranded overhanging ends in the cMycG·IS duplex.

Based on these collective observations, we propose that the formation of the G4·C4 loop-duplex domain by the strand invasion of the IS oligomer into the G4 quadruplex, flanked by upstream and downstream duplex arms, is a very slow process (2 weeks of incubation time at 4 °C) and at least partially is inhibited by the G4 quadruplex and the IS iDNA secondary structures. The slow formation of the G4·C4 loop-duplex at 4 °C suggests that the G4·C4 loop-duplex, while kinetically inhibited, is thermodynamically favored over the isolated IS iDNA and G4 quadruplex conformers. Support for this interpretation comes from our observation that the CD spectrum measured immediately after mixing can be approximated by the sum of the CD spectra of the components that were mixed together (cMycG·22merC0 and the IS), suggesting minimal interactions between the components that were mixed. By contrast, the CD spectrum measured after 2 weeks of incubation at 4 °C is best approximated by the sum of the CD spectra of the duplex components (22mer duplex and cMycG·IS minus 22merG0), suggesting that the conformation after 2 weeks of incubation includes interactions between the IS and cMycG·22merC0 that are substantially similar to the G4·C4 duplex seen in cMycG·IS (Figure 5).

In summary, based on the collective observations reported here, our data reveal a balance between slow strand invasion and the faster expulsion of the invading strand, as illustrated pictorially in the summary flowcharts. Such an intriguing balance of coupled events explains the melting behavior we observe without prolonged incubation at low temperatures. We further posit that upon the melting of the IS secondary structure in the freshly mixed samples, strand invasion accelerates relative to the 4 °C sample. Nevertheless, the strand invasion reaction remains slow relative to the slow heating rate of these experiments, thereby resulting in the observed offset in the temperature (and time) axis of the 2Ap fluorescence change relative to the IS CD melting curve. We further propose, based on the melting behavior of the 2-week-incubated sample, that during the melting process of the freshly mixed sample, any temperature-induced increase in the rate of strand invasion to form the G4·C4 loop-duplex is increasingly counteracted by the concurrent melting of some fraction of the newly formed G4·C4 loop-duplex.

Note that the initial melting of the cMycG·22merC0: IS sample that was incubated for 2 weeks starts and ends at a slightly higher temperature than the melting/invasion by the IS of the freshly mixed cMycG·22merC0:IS sample. The net result of these opposing processes, namely, the relatively slow invasion of the denatured IS into the G4 quadruplex to form the G4·C4 loop-duplex domain versus the relatively fast melting of the G4·C4 loop-duplex domain, yields the formation of a non-interacting cMycG·22merC0 complex plus the denatured IS construct following the completion of the first transition observed in the complex melting profiles presented above.

**Scheme 8:**
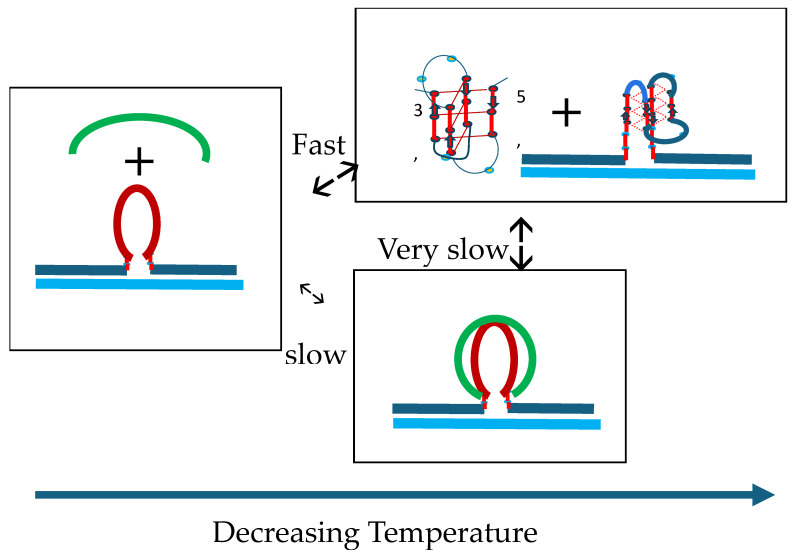
cMycG·22merC0 plus the IS hysteresis.

- Hysteresis in cooling curves reflects competing kinetic processes that occur at different relative rates and lead to the population of metastable intermediates, Scheme 8.

Our experimental results reveal hysteresis when reannealing/cooling the cMycG·22merC0:IS sample from temperatures above 35 °C. Specifically, our measurements display characteristic and reproducible features in the reannealing curve: namely, an initial slow decrease in fluorescence followed by a subsequent gradual increase in fluorescence at lower temperatures. The initial (slow) decrease in the fluorescence signal upon cooling from 35 °C to 20 °C is expected, given the slow rate of formation of the G4·C4 loop-duplex from the cMycG·22merC0 complex and the denatured IS. Indeed, incubation at 20 °C results in the slow mono-exponential kinetics of the formation of the invaded cMycG·22merC0:IS complex, while also exhibiting the spectral characteristics of the 2-week-incubated samples of cMycG·22merC0:IS when the reaction has reached its equilibrium state.

How, then, does one explain the gradual increase in fluorescence at temperatures below 20 °C in the annealing curves? The close inspection of the CD spectra obtained below 20 °C reveals an increase in that part of the CD signal that can be assigned to the iDNA form of the IS. The folding of the IS is an intramolecular process that is faster than the intermolecular interaction with the (folded) G4 domain. We also note that the melting of the IS native state is incomplete at temperatures below 20 °C. Since the IS does not contain 2Ap, the IS iDNA formation does not, by itself, cause an increase in the 2Ap fluorescence signal at lower temperatures. Rather, as discussed above, the rate of G4·C4 loop-duplex formation from the folded form of IS is slowed down significantly, thereby reducing the rate by which cMycG·22merC0 converts to the low fluorescence cMycG·22merC0:IS complex. The fluorescence of 2Ap in the center of a parallel-stranded G4 quadruplex shows a pronounced linear temperature dependence, even without gross conformational changes. In part, the increase in 2Ap fluorescence we observe below 20 °C can be assigned to this temperature dependence of 2Ap fluorescence in the parallel G4 quadruplex form that has not yet reacted with the IS.

Furthermore, as we discuss in subsequent sections, the unfolded IS appears to remain loosely associated with, but not formally bound to, the G4 loop domain, resulting in the quenching of 2Ap fluorescence in the G4 quadruplex. The addition of the folded IS at low temperatures, however, does not cause a similar 2Ap quenching prior to the strand invasion, suggesting that refolding of the IS into its iDNA conformation in competition with the strand invasion also reduces the IS-induced quenching of the cMycG·22merC0 complex, which contributes to the observed increase in 2Ap fluorescence.

Based on these observations, we propose that upon reannealing we observe temperature-dependent competition between the intramolecular refolding of the IS into the iDNA conformation in competition with the intermolecular strand invasion to form the G4·C4 loop-duplex. The relative fractional occupancies of these competing states depend on the relative rates of formation at each temperature interval, with the intramolecular iDNA formation initially outcompeting the strand invasion. The 2-week-incubation data, however, suggest that the invaded complex is thermodynamically the most stable state and eventually will form when sufficient time has passed (e.g., 2 weeks at 4 °C). This reannealing behavior we observe represents an example of how relative differences in rates of competing pathways can lead to metastable intermediates and dictate the fractional occupancies one observes (see also von Hippel) [6].

The different processes contributing to changes in the low-temperature heating and cooling curves of the cMycG·22merC0 plus the IS mixtures discussed above are summarized schematically in Table 2.

The inspection of the information embedded in each of the four top-to-bottom horizontal transformations illustrated in Table 2 leads us to the following conclusions:

(1) For a freshly prepared sample, we propose coupled transformations that correspond to the invasion of the G4 quadruplex by the IS, likely via an unfolded intermediate, followed, at slightly higher temperature, by the melting of the G4·C4 loop-duplex.
(2) For a sample incubated at 4 °C, we propose the formation of the G4·C4 loop-duplex, with the unfolded IS as a likely intermediate, with coupled transformations that collectively exhibit complex kinetics.
(3) For a sample incubated at 20 °C, we propose the formation of the G4·C4 loop-duplex with slow, single, exponential kinetics associated with the IS invasion
(4) For a sample preincubated at 4 °C, we propose the melting of the G4·C4 loop-duplex to form cMycG·22merC0 and the IS. However, as we show in the next section, the IS remains closely associated with, but not formally bound to, cMycG·22merC0, leading to altered the properties of the cMycG·22merC0 complex.

#### 3.3.3. Temperature Domain [II.] cMycG·22merC0 in the Presence of the IS-Coupled Transformations, as Illustrated and Discussed Below

**Scheme 9:**
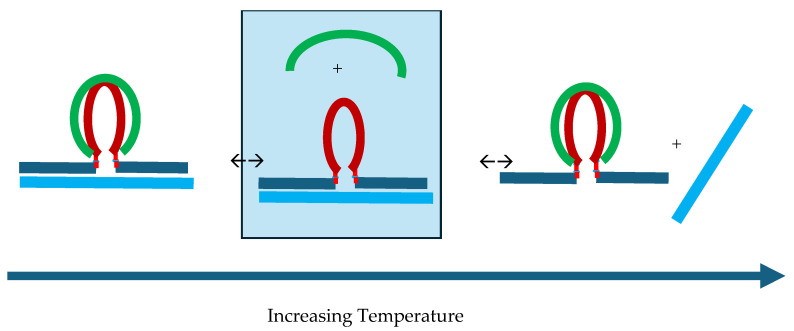
cMycG·22merC0:IS; the coupled exchange of the IS and 22merC0 bound to cMycG.

- The comparison of “cMycG·22merC0:IS” with “cMycG·22merC0”: evidence for IS-induced changes in the nature of the G4 loop domain, Scheme 9.

In Figure 6, we compare the fluorescent melting curve of the isolated cMycG·22merC0 complex (blue curve) to those observed for the 1:1 cMycG·22merC0:IS mixture at different incubation times. To facilitate this comparison, we scaled the y axis of the isolated cMycG·22merC0 melting curve in Figure 6 to mimic the magnitude of the cMycG·22merC0:IS transition that it most closely resembles in intensity. Note that we observe close agreement between the isolated cMycG·22merC0 melting transition and the 2nd, reversible, transition (transition 3 in Figure 3) at around 48 °C, as seen in all the cMycG·22merC0:IS samples, regardless of the prior history of the sample. This observation suggests that the low temperature transitions (i.e., the transitions labeled 1 and 2 in Figure 3—see also Table 2) of the cMycG·22merC0:IS complex indeed result in the formation of the cMycG·22merC0 complex plus the denatured IS strand. Subtle differences in the shape of the second melting transition (transition 3 in Figure 3) of cMycG·22merC0:IS relative to cMycG·22merC0 on its own, primarily on the high temperature side of the melting transition, are indicative of the impact of the IS on cMycG·22merC0. Specifically, as outlined in Table 3, these subtle differences in the shape of the melting curve relative to that of cMycG·22merC0 without the IS reflects the binding of the IS to free cMycG after the dissociation of 22merC0, in what amounts to a coupled exchange reaction. We further discuss this exchange process below in the section on the melting domain [III.].

- cMycG·22merC0 in the presence of the free IS differs from isolated cMycG·22merC0 without the IS.

**Figure 8:**
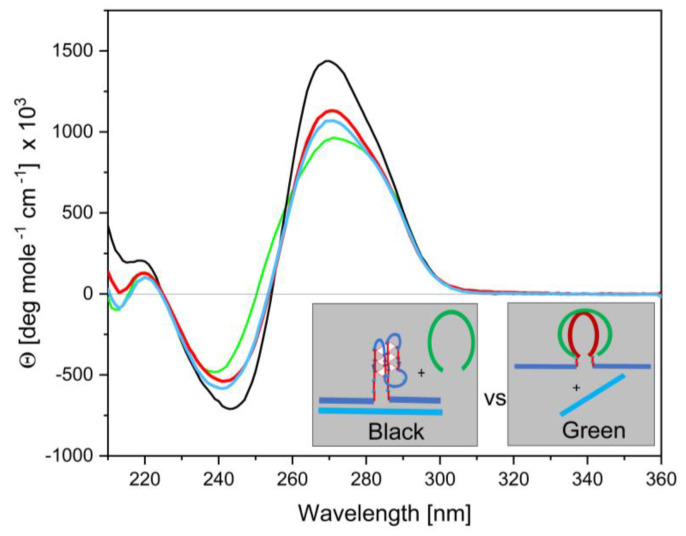
Comparison between CD spectra for the cMycG·22merC0:IS complex at 36 °C (red: initial heating; blue: following 2 weeks of incubation) versus calculated CD spectra derived by the addition of the IS spectrum at 36 °C to native cMycG·22merC0 in K+ (black) versus the spectrum of isolated 22merC0 36 °C added to the spectrum of cMycG·IS (green). The lack of agreement in the CD spectra compared in Figure 8, especially in terms of the iso-elliptic points between the measured CD spectra and the calculated/expected spectra for cMycG·22merC0 plus the IS and cMycG·IS plus 22merC0 is consistent with the cMycG·22merC0:IS sample at 36 °C not being reflective of a mixture of these two species.

Interestingly, when we compare the fluorescence melting curves of cMycG·22merC0:IS and cMycG·22merC0 in real terms (i.e., not scaled to reflect similar changes in the magnitude of the fluorescent change in the transition region) we find significant differences in 2Ap fluorescence intensity following the expulsion of the IS strand at temperatures above ~35 °C, compared to an cMycG·22merC0 sample that was never exposed to the IS, despite identical sample concentrations. As illustrated in Figure 7, at temperatures below 10 °C and above 65 °C, the 2Ap fluorescence intensities in both samples are almost identical, but between 10 °C and 65 °C there are significant differences. These fluorescence intensity differences also are mirrored by subtle differences in the corresponding CD spectra in this temperature range. (Figure 8). A direct comparison of the fluorescent melting curves in absolute terms (i.e., in “real terms”) is justified, as the signal in both cases originates from the single 2Ap base in the cMycG strand that is present in identical concentrations in both samples. A small reduction, not exceeding 6% in the total fluorescence of the IS-containing sample, can be assigned to an inner filter effect caused by the non-zero absorbance at 308nm of the added native IS iDNA. Such an inner filter effect is expected to diminish as the IS melts/binds to the G-quadruplex loop, since neither the bound nor denatured IS retains significant absorbance at 308 nm compared to the iDNA state of the IS. The inner filter effect, however, cannot explain the observed loss of fluorescence seen in the presence of the IS relative to the IS-free sample at ~35 °C. We note that the measured CD spectrum of the cMycG·22merC0:IS mixture at 36 °C cannot be simply explained in terms of the sum of the CD spectra of isolated cMycG·22merC0 and the free IS (black line in Figure 8). Collectively, these comparative observations suggest that after the expulsion of the invading IS strand, either the IS, or cMycG·22merC0, or both adopt different CD-sensitive conformations relative to the isolated components.

Equally intriguingly, we find that when the IS is added to preformed cMycG·22merC0 at 36 °C, a temperature above the first melting transition of the cMycG·22merC0:IS complex, we observe a rapid loss of fluorescence intensity and a change in the CD spectrum to values similar to what is seen during the melting of the cMycG·22merC0:IS complex, as previously noted. The lack of further changes in the time-dependent spectral properties of a preformed cMycG·22merC0:IS sample heated to 36 °C suggests that an equilibrium state is reached after the first melting transition. How, then, does one explain the loss in fluorescence for the cMycG·22merC0:IS complex between 35 °C and 55 °C, when the invading IS strand has been expelled? In the next section we propose a possible explanation for these intriguing observations.

- The IS remains loosely associated with cMycG·22merC0 without being bound in a formal sense.

To explain these observations, we favor a model wherein the IS strand expelled from the G4·C4 loop-duplex domain remains loosely associated with the cMycG·22merC0 complex, without being formally bound via conventional base-pairing interactions. Such loosely associated DNA states are reminiscent of solvent-separated ion pairs, which also are loosely associated as opposed to formally bound, like condensed counterions, as compared with the corresponding solvent-excluded, intimately “bound” ion pairs [127,128]. Based on our analysis, we posit that such an “IS associated state” may well reflect an IS-induced altered equilibrium between a planar and a folded state of the pseudo three-way junction formed by the parallel G-quadruplex embedded between the upstream and downstream duplex domains. DNA three-way junctions containing unpaired bases at the junction [134,135,136,137,138], as well as DNA pseudo-three-way junctions, in which one of the arms is a CAG repeat bulge loop [139,140], are known to exist in a salt-dependent dynamic equilibrium between a planar and one or two related folded states that differ by the relative stacking arrangement between the three arms in 3-dimensional space. This condition can prevail without altering base-pairing interactions at the junction. Evidence for planar and bent/folded forms for extruded G-quadruplexes embedded within a larger duplex domain without an invading strand, but conceptually similar to the one we have studied here, have recently been described based on cryo-microscopy studies [141]. For these collective reasons, it is not unreasonable to suggest that the addition of the polyanionic IS strand, which is “associated with the G quadruplex DNA”, may well alter such an equilibrium without necessarily being formally bound to the G-quadruplex domain. Notably, changes in a planar vs. the folded equilibrium of the G-quadruplex pseudo-three-way junction due to a loosely associated, but not formally bound, IS strand is consistent with the observed subtle CD changes and more pronounced fluorescence changes described above. Moreover, such a “loosely associated”, but not formally bound, state may well reflect an early transition state along the pathway of the strand invasion. Such a pathway would be reasonable to consider if and when conditions (e.g., temperature) allow for the strand invasion to either form the G4·C4 loop-duplex upon cooling or to form the G4·C4 duplex upon the expulsion of the 22merC0 strand.

#### 3.3.4. Temperature Domain [III.]. The Formation and Melting of the cMycG·IS Complex in the Presence of 22merC0

**Scheme 10:**
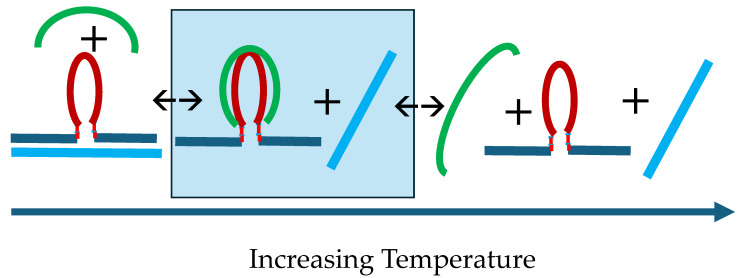
cMycG·22merC0 plus IS <-> cMycG· IS plus 22merC0 <-> cMycG plus IS plus 22merC0.

- The comparison of “cMycG·22merC0:IS” with “cMycG·IS”: evidence for a reversible, temperature-controlled switch between cMycG·22merC and cMycG·IS, Scheme 10.

**Figure 9:**
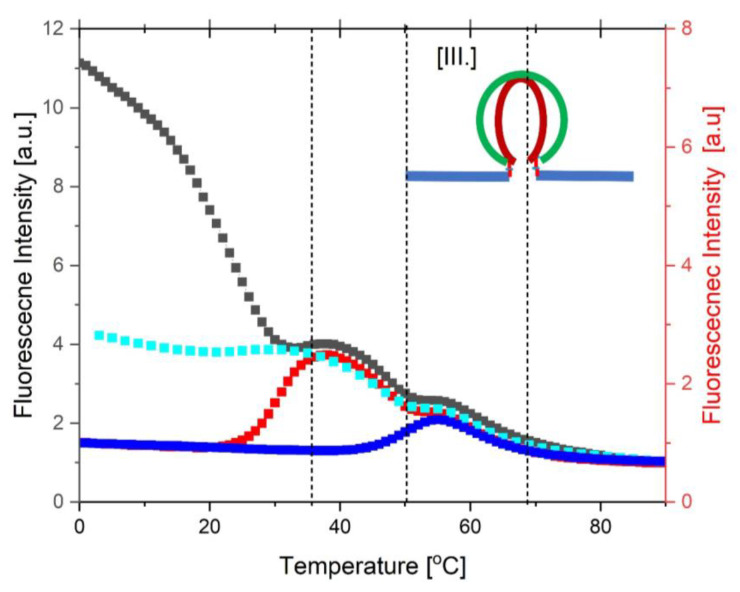
An overlay of the fluorescent melting curves measured for the isolated cMycG·IS complex (blue curve) and the fluorescent melting curves for the 1:1 mixture of preformed cMycG·22merC0 with the IS immediately after mixing (black curve) and after incubation for 2 weeks at 4 °C (red curve). The reannealing curve is shown in cyan. The inspection of Figure 9 reveals a close agreement between the high temperature transitions in all three melting curves. This concurrence suggests that the cMycG·IS complex also is present in the samples consisting of cMycG, 22merC0, and the IS at high temperatures, despite the evidence that the IS is expelled from the G4·C4 loop-duplex at lower temperatures (i.e., see prior discussion of reaction 2).

**Figure 10:**
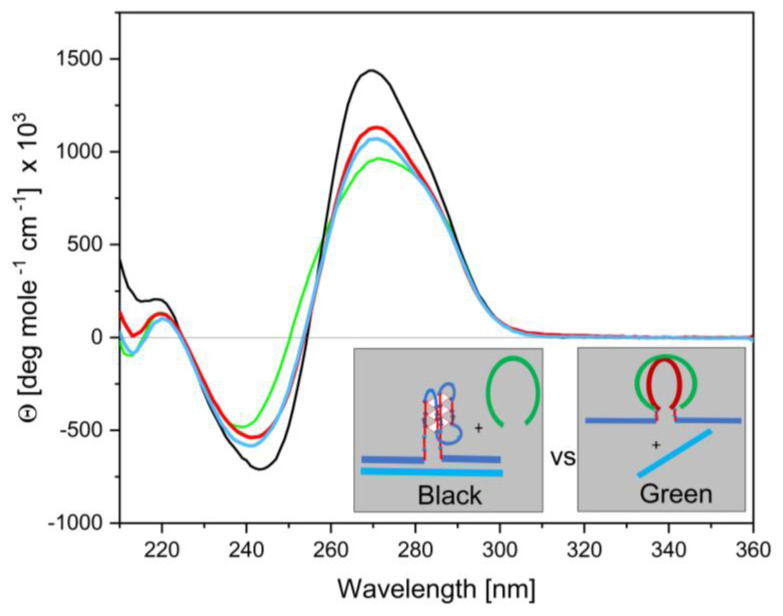
The comparison of the observed CD spectrum for cMycG·22merC0:IS at 50 °C (red: initial heating; blue: 2 weeks of incubation) versus the calculated CD spectra by the addition of the 22merC0 spectrum at 50 °C to that of native cMycG·IS at 50 °C (black).

Figure 9 compares the third melting transition observed for all the cMycG·22merC0:IS samples (transition 4 in Figure 3) with the fluorescent melting curve of an isolated cMycG·IS duplex. Recall that we previously proposed that the reversible transition, 3, corresponds to the melting of cMycG·22merC0 in the presence of the “free” IS. However, given that the isolated cMycG·IS complex exhibits a higher melting temperature than cMycG·22merC0, the denaturation of cMycG·22merC0 allows for the formation of cMycG·IS from the newly liberated and fully unfolded cMycG strand and the free IS strand that is already present in the solution. The coincidence in the melting curves observed for the third melting transition of the cMycG/22merC0/IS sample with the melting curve of the isolated cMycG·IS duplex suggest that, upon the melting of cMycG·22merC0, cMycG reassociates with the IS to form cMycG·IS with free 22merC0. In effect, melting transition 3 corresponds to a coupled exchange of DNA strands bound to cMycG from 22merC0 to the IS. The confirmation of this assignment comes from a comparison of the measured CD spectra at 50 °C with the CD spectra calculated by adding up the independently measured spectra for cMycG·IS and free 22merC0, as illustrated in Figure 10.

- Temperature controlled reversible exchange between 22merC0 and the IS bound to cMycG.

More interesting than the initial formation of the cMycG·IS complex upon the melting of cMycG·22merC0 and its subsequent denaturation is the observation that both transitions are fully reversible upon cooling. This observation suggests that during cooling from the fully denatured state, cMycG·IS initially reforms in accordance with its higher melting temperature, while the 22merC0 remains free in the solution. However, at the temperature where the cMycG·22merC0 complex in isolation becomes stable, the 22merC0 strand displaces the already-bound IS strand to reform the cMycG·22merC0 complex, releasing the bound IS strand. We have tested this hypothesis by adding free 22merC0 to a preformed cMycG·IS complex at 36 °C. Based on the characteristic CD and fluorescence changes, we observe the rapid displacement of the bound IS strand to form cMycG·22merC0 and the “free” (perhaps “loosely associated”) IS. As noted earlier, this observation is consistent with our melting studies, which suggest that by 36 °C, IS has been displaced from (but perhaps remains loosely associated with) the cMycG·22merC0 complex, thereby resulting in cMycG·22merC0 plus the “free” IS.

The rapid displacement of the IS strand suggests that the overhanging upstream and the downstream single-stranded arms in cMycG·IS provide ideal “toehold-like” nucleation sites [142,143,144] for initiating cMycG·22merC0 formation. However, our system is unlike the ‘conventional’ toehold strand displacement reactions, where the invading strand binds to a single-stranded toehold domain and then displaces an already-bound strand downstream from the toehold domain by competition for base-pairing interactions. Instead, in our case, the 22merC0 does not physically compete with the IS strand for base-pairing sites, but rather bridges the G4 domain to optimize all the base-pairing interactions in the upstream and downstream duplex domains. Simultaneously, the base-paired G4·C4 domain is forced to loop out. We posit that constraints imposed by the persistence length of G-rich duplexes that make up the G4·C4 loop-duplex, when forced to conform to the restricted loop dimensions, destabilize the base-pairing interactions between the G4 domain and the IS strand. We posit further that such destabilization of the base-paired loop region, coupled with the availability of energetically favorable intra-loop G-G type base-pairing interactions within the single-stranded G4 loop, collectively result in the displacement of the IS strand at intermediate temperatures (i.e., temperature domain [II.]). We also know from the results we presented above that at much lower temperatures (i.e., in temperature domain [I.]), the IS can successfully reinvade and form a stable G4·C4 loop-duplex complex, albeit one with a much lower melting temperature and very slow formation kinetics. These observations suggest significantly different temperature dependencies in the balance of the forces stabilizing/destabilizing the G4·C4 base pairs within a linear duplex versus within a loop-duplex arrangement. Such balancing of competing forces may be required for the invading strand to act as a catalyzme/catassembler ([145,146]). Additional support for such a novel point of view comes from our observation that in a dumbbell construct, which is less able to accommodate a base-paired rigidly constrained G4·C4 domain due to restricted rotation of the upstream and downstream duplex domains, the melting temperature for the displacement of the invading IS strand is even lower than in the **cMycG·22merC0:IS** complex reported here (unpublished results), while the G4·C4 duplex with unpaired upstream and downstream duplex arms is never populated.

Table 4 schematically depicts the conformational transitions associated with reaction 4 (Figure 3) in temperature domain [III.]

### 3.4. How the IS Interacting with the G4 Quadruplex Domain Influences cMycG·22merC0 Properties

#### 3.4.1. The Invasion of the G4 Quadruplex Is Kinetically Inhibited by the Quadruplex Fold, as Well as by the Self-Structure of the Invading Strand

To summarize, we find that the IS, when added to the preformed cMycG·22merC0 quadruplex, can unfold and invade the parallel-stranded G-quadruplex domain of cMycG·22merC0 at low temperatures to form a constrained G4·C4 loop-duplex embedded within the upstream and downstream duplex domains, although it does so very slowly. The main conclusions discussed in this section are pictorially summarized in Chart 2. The G4·C4 loop-duplex that results from the invasion process has features resembling that of a three-stranded, branched D-loop, a feature that plays important roles at telomere ends and during nonhomologous strand exchange processes [13,14,19,125,126,147,148]. The complex invasion kinetics at 4 °C, where the IS adopts the iDNA conformation, coupled with the more conventional, but slow, single exponential kinetics at 20 °C, where the IS is fully unfolded, suggest that the invasion process proceeds via the unfolded state of the IS. However, even at 20 °C, where the secondary structure of the IS is fully denatured under our conditions, the kinetics of the strand invasion are still orders of magnitude slower than expected for conventional duplex formation [149,150,151]. We also find very slow kinetics for the G4·C4 duplex formation compared to non-quadruplex forming sequences for interactions of denatured IS with the cMycG single strand. The G4 domain within the cMycG single strand adopts an ensemble of G-quadruplex conformations that includes, among other, the parallel G-quadruplex adopted by cMycG·22merC0. We also find that addition of 22merC0 to a preformed cMycG· IS duplex already containing the G4·C4 duplex at low temperatures (20 °C and below) results in the comparatively rapid formation of the cMycG·22merC0:IS complex, which is seen only after two weeks incubation when adding the IS to preformed cMycG·22merC0.

Considering these multiple observations/results collectively suggests that, in addition to the IS self-structure, the quadruplex fold also slows down the invasion process. It is likely that the base-pairing arrangements in the G-quadruplex inhibits the initiation complex formation with (unfolded) the IS, thereby slowing the invasion kinetics despite the resulting duplex being the thermodynamically more stable state. We also posit that the kinetics of the denatured IS invasion is slowed down by the sequence redundancy inherent in any G-quadruplex domain, (as well as the invading complementary strand) which can lead to sequence misalignment in some of the initiation complexes. Given the cooperative nature of the G-quadruplex structure, we expect that even a misaligned initiation complex formation between the invading IS strand and the G4 domain will disrupt the quadruplex structure. While such misaligned initiation complexes will need to be unfolded again for the strand invasion to proceed to completion, it may be sufficient to prevent the refolding of the G-quadruplex on the timescale of the “repair” of the misaligned initiation complex. The combination of both processes, in addition to the need to, at least partially, unfold the invading IS strand, makes for a highly “rough” energy landscape for invasion, with many local energy minima traps, thereby resulting in the correspondingly slow invasion kinetics. Going forward, it may be of interest to see if invasion by a DNA strand that is able to form a toehold “guide duplex” [144], with bases of one of the adjacent duplex domains, might speed up the invasion kinetics, not only through improved initiation complex formation via the toehold domain but also by guiding the alignment of the invading strand.

#### 3.4.2. The Product of the Invasion of the G-Quadruplex by the IS: The G4·C4 Loop-Duplex Is Thermally Less Stable than the Corresponding Linear G4·C4 Duplex

Once formed, the G4·C4 loop-duplex is relatively unstable compared to the equivalent G4·C4 duplex that is not constrained into a loop topology by neighboring duplex arms. The constraint imposed by the loop topology leads to a decrease in the melting temperature of some 30 °C (ΔTm = 30 °C) relative to the unconstrained duplex, as summarized in Chart 4. Interestingly, the expulsion of the invading IS strand does not lead to the recovery of the optical properties characteristic of the parallel G4 quadruplex structure in cMycG·22merC0, even though the parallel G4 quadruplex is stable at this temperature without the IS strand.

#### 3.4.3. The Expulsion of the Invaded IS Strand Does Not Allow for the Refolding of the G4 Quadruplex into the Conformation It Adopts Without the Invading Strand

The subtly different CD spectra and vastly different fluorescence intensities of cMycG·22merC0 with and without the IS present are consistent with structural alterations in the cMycG·22merC0 quadruplex after the expulsion of the invading IS strand. It is currently unclear what conformation is adopted by the G4 domain in cMycG·22merC0 in the presence of the IS, except that it is clearly not a fully unfolded loop either, based on a comparison to cMycG·22merC0 in Li salt. The change in the nature of the G4 domain is also not related to a trapped conformation after the expulsion of the invaded IS strand, as it also forms rapidly when the IS is added to the fully folded cMycG·22merC0 at temperatures above the expulsion temperature for the IS and below the melting temperature for cMycG·22merC0. Further, we do not detect any melting transition that could be assigned to partial base-pairing interactions between the IS and the G4 domain, although such a transition, if it exists, may be masked by the other melting processes. We speculate that the presence of the IS strand might alter the open (planar)/closed (two stacked arms) equilibrium of the pseudo-three-way junction that a G-quadruplex embedded within the upstream and downstream duplex domains resembles [135,136,137,138,140,141]; however, we currently do not have experimental evidence for this assertion. In the absence of additional information, we have chosen to refer to the conformation adopted by cMycG·22merC0 in the presence of the IS as an “IS associated” conformation. It will be of considerable interest to see to what extent this “IS associated” state differs in terms of thermodynamic and kinetic stability compared to the parallel-stranded G4 quadruplex without the IS strand. Furthermore, it would be of interest to explore if and how the interactions of G4 quadruplex processing enzymes are altered by the presence of an IS or related oligonucleotide strands. Possible changes in regulatory outcomes due to subtle differences in the DNA topology not requiring altered base-pairing arrangements, perhaps triggered by the presence of an “associated” DNA strand, similar to what we observe here, have heretofore not been widely considered.

#### 3.4.4. The IS Displaced from the G4·C4 Loop-Duplex Domain Can Reversibly Rebind cMycG After the Melting of the cMycG·22merC0 Complex

We further observe that when cMycG·22merC0 melts in the presence of the IS, the IS rebinds to the now-free cMycG strand to form the cMycG·IS duplex with overhanging 3′ and 5′ single strands. The melting of cMycG·22merC0 and the formation of cMycG·IS is a coupled process driven entirely by the differential stabilities of the base-pairing interactions involved. It is of further interest to note that the coupled melting/formation reaction is entirely reversible upon cooling, with no hysteresis. In other words, upon cooling, the preferred formation of cMycG·22merC0 at lower temperatures is coupled to the rapid displacement of the IS from cMycG·IS, an observation we also have confirmed by adding a free 22merC0 strand to preformed cMycG·IS within temperature domain [II.]. Intriguingly, the highly G-rich G4·C4 domain does not provide a kinetic block to the strand displacement by the 22merC0 strand. The strand displacement of the IS by 22merC0 is entirely thermodynamically controlled and likely aided by the ability of 22merC0 to rapidly form stable “toeholds” with either the upstream or downstream arms of cMycG, or both. These observations provide yet another manifestation of an energetic cost being imposed by the loop topology on the G4·C4 duplex (see also Chart 4). The loss of the base-pairing interactions between the G4 domain and the IS is paid for by optimizing base pair formation in the upstream/downstream duplex arms, thereby constricting the G4 domain to a loop topology coupled with the self-structure formation within the free G4 domain, even though the linear G4·C4 duplex formed between cMycG and the IS is thermodynamically stable under these conditions. The fact that IS can and does bind to the G4 domain again at much lower temperatures (<30 °C) suggests significant differential heat capacity influences in this system that may not be readily apparent, based on the knowledge of the heat capacity terms of its linear components [152,153,154,155,156,157,158]. It is worth noting that such differential temperature/heat effects coupled with topological constraints are likely an important, and largely unexplored, feature of higher-order nucleic acid structures, especially those that can exist in a dynamic equilibrium between different conformational states.

#### 3.4.5. The Hysteresis upon Cooling Is Driven by Differential Rates of Interactions Between the Invasion Complex and the IS Self-Structure

As summarized in Chart 3, the results described above allow us to understand the hysteresis observed in temperature domain [I.] upon cooling. It is precisely the preference for the cMycG·22merC0 duplex over the cMycG·IS duplex that creates the conditions that lead to the hysteresis we observe. To be specific, we have shown that the addition of 22merC0 to preformed cMycG·IS at low temperatures leads to the rapid formation of the cMycG·22merC0:IS complex, but the addition of the IS to preformed cMycG·22merC0 requires a 2-week incubation time to achieve the same result. The preferential formation of cMycG·22merC0 upon cooling means that at the boundary between temperature domains [II.] and [I.], at ~30 °C, the sample contains the unfolded IS that is in some manner “associated”, but not formally bound in a classic base pairing sense, with cMycG·22merC0. The G4 domain in the cMycG·22merC0 complex formed upon cooling in the presence of the IS is structured, but not necessarily in its equilibrium parallel G4 quadruplex fold. The exact nature of the G4 domain and its “association” with the IS is, as yet, unknown; however, the detailed knowledge of the specifics of this “soft” interaction is not needed for understanding the basis of the hysteresis phenomenon. The invasion of the G4 domain by the unfolded IS oligonucleotide occurs, but it proceeds more slowly than the change in temperature during our cooling experiment, with the invasion rate slowing down further as the temperature decreases. Below 20 °C, a competing reaction in the form of the much-faster intramolecular folding of IS into the iDNA state becomes more pronounced. iDNA formation by the IS is primarily thermodynamically controlled, meaning the fraction of the denatured iDNA that folds into the iDNA state is determined by the temperature, at least for the relatively slow cooling rates studied here. It is worth underscoring that DNA constructs with longer C tracts folding into the iDNA state can also become dependent on time [159,160]. The iDNA folded state of the IS, however, is thermodynamically less stable than the IS when base-paired to the G4 domain of cMycG·22merC0. Consequently, below 20 °C, the initial fractional occupancy of the iDNA state and the cMycG·22merC0:IS complex is determined entirely by the difference in rates by which these two states form, with the iDNA state, once formed, gradually unfolding and invading the cMycG·22merC0 complex at a very slow rate. In short, as the temperature is lowered below 20 °C, the much faster rate of forming the iDNA state relative to the cMycG·22merC0:IS complex leads to the fraction of unfolded IS to preferentially fold into the iDNA state, which represents a kinetically trapped metastable state. The similarity in the cooling curves for both samples shown in Figure 2 and/or Figure 3 reflects the identical cooling rates from identical initial conditions at 30 °C. Significantly, under such circumstances, the dominant factor that determines the population distribution upon annealing is time, rather than thermodynamic stability.

### 3.5. Integrating and Summarizing the Interactions Between the Multiplicity of DNA States

The primary results of our analysis of the multiple conformational transitions discussed here are summarized pictorially in Chart 1. We initially presented this flowchart near the beginning of this article to foreshadow and summarize the time and temperature-dependent interacting DNA states proposed herein, based upon differential spectroscopic profiles. We reproduce it again here, as well as the other flowcharts, so we can further elaborate on the assigned transformations, now in the context of the more-detailed spectroscopic characterizations that we subsequently presented.

**Chart 1:**
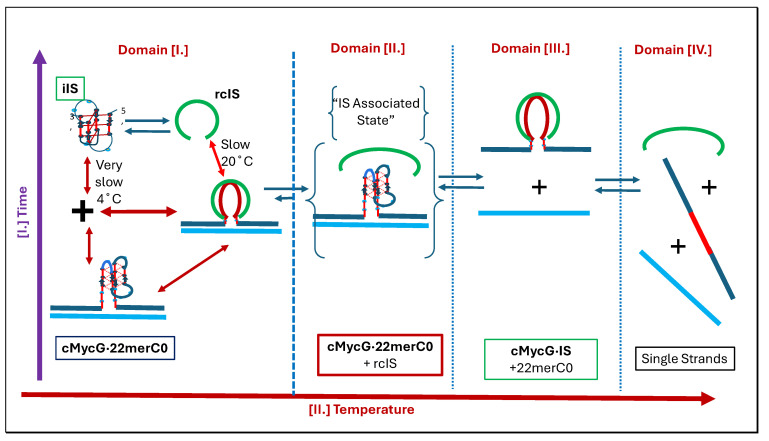
Flowchart 1.

Our collective results are consistent with three overarching conclusions, which we propose reveal how coupled and uncoupled transformations aid in our understanding of dynamic processes in higher-order nucleic acid systems. As we present each general conclusion below, we also underscore specific practical implications/applications. We emphasize that potential in vivo regulatory pathways may make use of the myriad of countervailing kinetically and thermodynamically controlled transformations and species distributions associated with the cascade of interconversions activated/triggered by the strand invasions of nonconical, higher-order DNA polymorphs. We list below our global conclusions, as well as, when appropriate, pictorially illustrate the associated molecular event(s) within the relevant flowchart(s).

Chart 2: Strand invasion and disruption of secondary structure.

**Chart 2:**
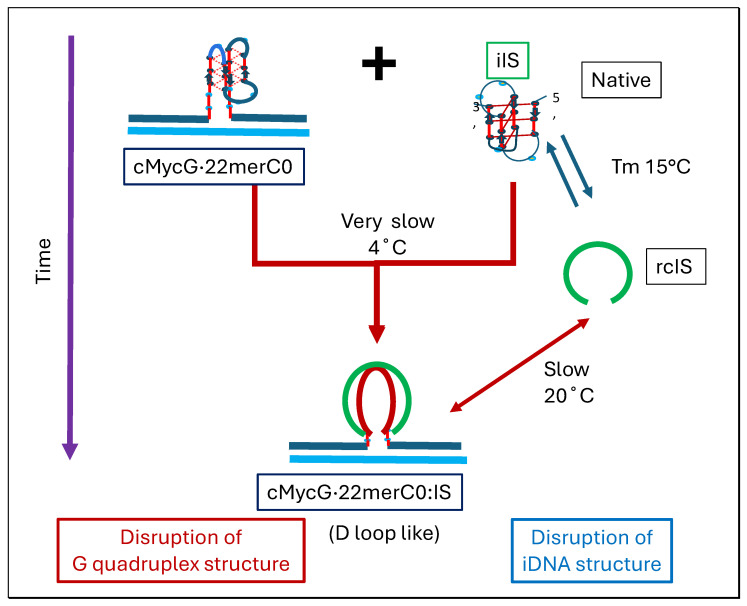
Flowchart 2.

(1) Depending on the temperature domain probed, a short C-rich oligonucleotide, in either its iDNA folded state or its unstructured denatured state, which possesses a sequence complementary to a parallel G-quadruplex strand, can invade and disrupt the DNA secondary structure of the target species (see flowchart). The invasion process also disrupts any secondary structure present in the invading strand. This overall process exhibits slow kinetics, reflective of the composite impact of the different initial states of the invading C-rich strand, as well as any coupled perturbations in the target G-quadruplex.

Such strand invasion and the disruption of DNA secondary structures, in either of or both the invading and invaded species, represent a potential pathway for the controlled unfolding of kinetically trapped DNA states, such as the parallel G-quadruplex studied here. The ability to unfold kinetically trapped states in a controlled manner is a feature that would enhance the ability for such states to be involved in biological regulatory mechanisms, such as have been proposed for the G-rich domain of the NHEIII element in the *cMyc* oncogene promoter, on which our model system is based [78]. It is also a potential avenue by which such DNA secondary structure-based regulatory switches may be manipulated for biomedical purposes via the deliberate addition of exogenous invading oligonucleotides. Our results suggest the use of such invading exogenous oligonucleotides, or oligonucleotide analogues, in a modified antisense/antigene strategy to regulate oncogene expression. More generally, the observations reported here suggest a potential avenue by which any DNA secondary structures susceptible to strand invasion events may be controlled and regulated.

(2) Our results also highlight the importance of metastability for noncanonical nucleic acid structures, as well as the interplay between slower and faster kinetic processes in determining preferentially populated states at any given point in time.

Furthermore, our spectroscopic mapping of higher-order DNA conformational transformations suggests that the population of specific states can be defined and modulated by the sample history, irrespective of where the thermodynamic equilibrium lays.

Chart 3: Metastability and sample history.

**Chart 3:**
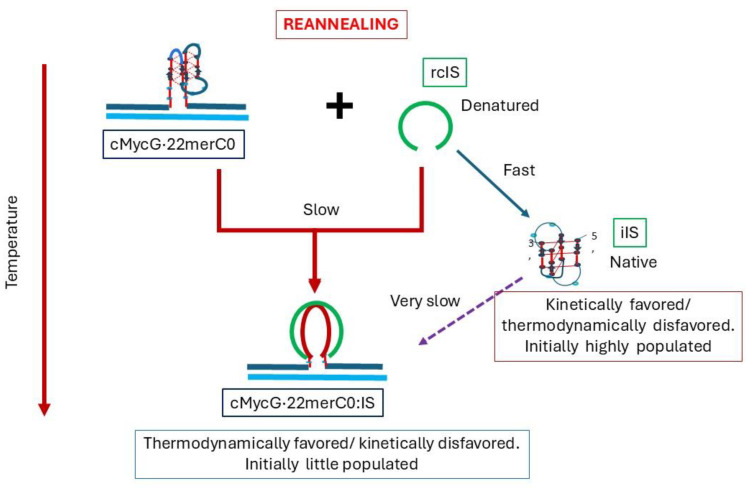
Flowchart 3.

This observation underscores the importance of the sample history in terms of relative refolding rates that dictate the populations of intermediate states, which, in turn, influences the preferred pathway for further folding steps, as revealed in our reannealing data (see Flowchart 3).

(3) Finally, our results provide evidence for topological constraints impacting the stabilities of base-paired domains, which, in turn, may impact the biological outcomes of higher-order nucleic acid secondary structures. Topological constraints are not readily accounted for in current nucleic acid models.

Chart 4: Topological constraint.

**Chart 4:**
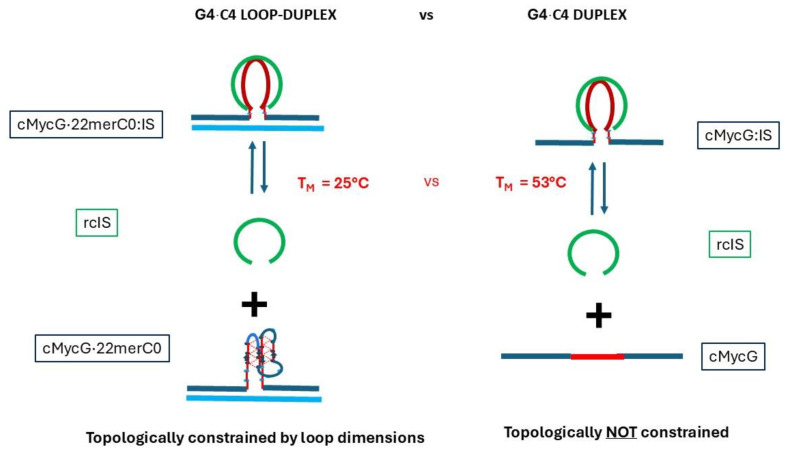
Flowchart 4.

We observe that the G4·C4 duplex formed between the G4 domain and the invading IS oligonucleotide can be disrupted at low temperatures when constrained by loop dimensions, as well as reversibly re-forming at higher temperatures when the loop constraint is absent. These observations provide a compelling example of the influences of topological constraints. Furthermore, topological constraints do not need to be large to exert their effects. Our results further suggest the impact of differential heat capacity effects, an influence that also is not readily accounted for in the current models of DNA rough energy landscapes.

In mapping the cascade of strand-invasion-induced DNA transformations, we primarily used temperature to manipulate the populations of different DNA oligonucleotide states. However, the transient formation of DNA states related to our temperature-induced states also can be induced isothermally as a result of several biological processes acting on genomic DNA. For example, strand separation as a result of the passage of a polymerase can potentially cause the formation of isothermal single-loop domains that, in turn, can interact with short invading strands like the IS. When the resulting loop is similar in size to the G4 domain and/or has folded into its competing G-quadruplex form, interactions between the short invading strand and the transiently single-stranded, looped-out domain are likely to be topologically constrained, as we observe here for cMycG·22merC0:IS. However, when the resulting loop is very large, interactions between the invading strand and single-stranded, looped-out domain are more like what we observe when the cMycG·IS complex is formed. In either case, an invading, short, IS-like oligomer can have pronounced effects on the resulting pathway by which the (transient) equilibrium is re-established.

## 4. Conclusions

In the aggregate, the spectroscopic studies reported here demonstrate the diversity and complexity associated with the mapping of the countervailing forces, and the coupled/linked transitions, that collectively dictate the relative populations and inter-related transformations of DNA species produced by strand invasion of noncanonical, higher-order DNA structures (e.g., G-quadruplexes and iDNA tetraplexes). Such higher-order structures are of particular interest, given their frequent over-representation in gene promoter and enhancer regions [28,60,61,62]. As we have underscored, the strand invasion of noncanonical, higher-order DNA secondary structures produces a range of DNA polymorphs that exhibit unanticipated properties that likely could be of functional significance. We also note that similar kinetically and thermodynamically controlled dynamic transformations, modulated by strand invasion events, and the associated rough energy landscapes are likely to be consequential for RNA, whose biological functions frequently are determined by dynamic transformation between higher-order folding isomers. Consequently, studies such as those reported here not only are important for a fundamental understanding of the diversity of the forces that modulate the “rough” energy landscapes associated with the kinetically and thermodynamically controlled polymorphism of higher-order DNA (and perhaps RNA) constructs, but also are essential for the rational design of ligands/drugs that selectively target biologically relevant check points within such complex conformational cascades induced by strand invasion events. After all, the most important DNA regulatory target(s) do not necessarily align with the most stable and/or the most highly populated DNA species. Hence the significance, as emphasized here, of mapping the “rough” energy landscapes of noncanonical DNA structures and their associated transformational pathways.

## Figures and Tables

**Chart 1:**
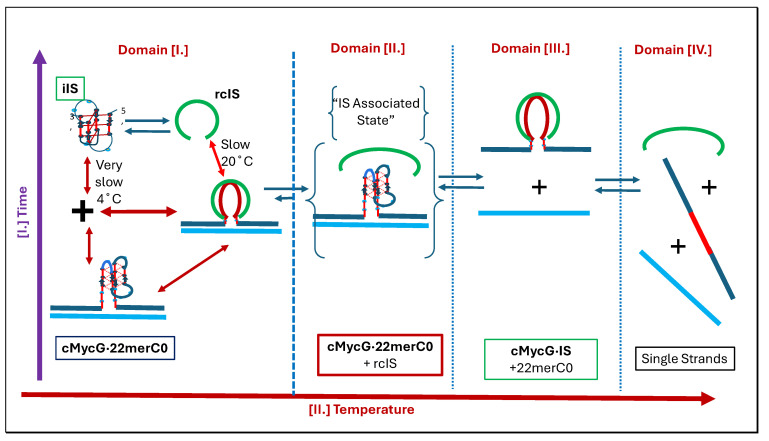
Flowchart 1.

**Chart 2:**
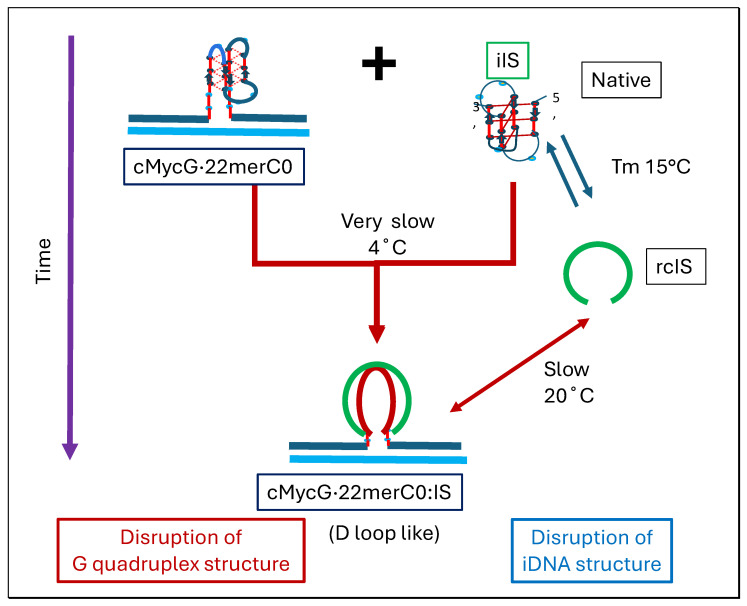
Flowchart 2.

**Chart 3:**
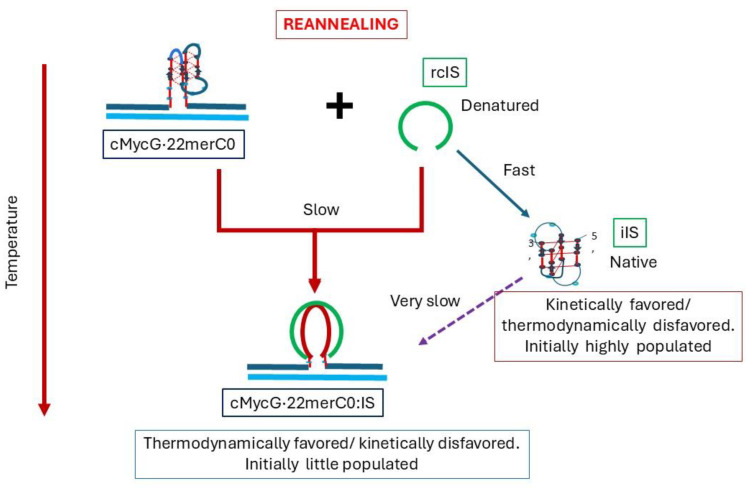
Flowchart 3.

**Chart 4:**
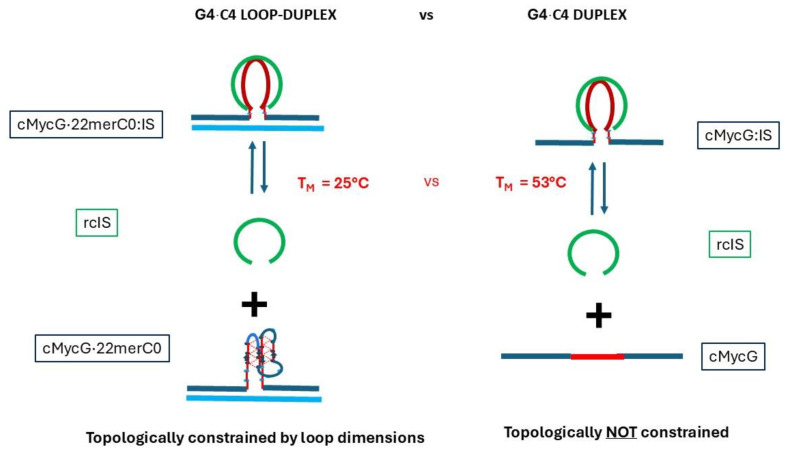
Flowchart 4.

**Table 1:** (**a**) Sequences of oligonucleotides used in this study; (**b**) pictorial representation of the various DNA states that can result from different combinations of the oligonucleotides listed in (**a**).

**(a)**			
**Name**	**Sequence**		**Construct Type**
cMycG	5′-CAC GAG CAC GC TTG GGA GGG T/2Ap/G GGA GGG TT CA CAC CGA AGC-3′		Duplex construct containing an embedded G-quadruplex domain
22merC0	↓ 5′-GCT TCG GTG TG GC GTG CTC GTG-3′		
IS	5′-CC CTC CCTACC CTC CC-3′		Invading strand
**(b)**			
**Name**	**Single Strands**	**Complexes**	**Complex Name**
22merC0	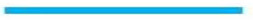	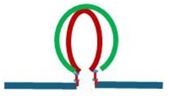	cMycG·IS
cMycG	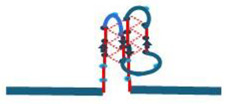	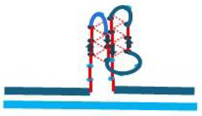	cMycG·22merC0
IS	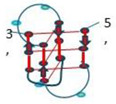	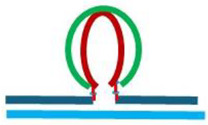	cMycG·22merC0:IS

**Table 2:** Schematic outline of time and temperature dependent processes within temperature domain [I.].

Domain [I.] 0 °C < T < 35 °C	Reaction	Comments
[I.] Fresh sample	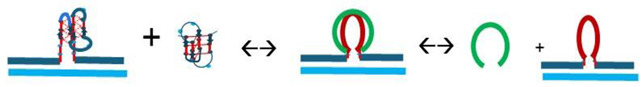	Temperature
[I.] Incubation @ 4 °C	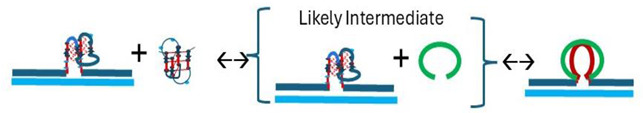	Time
[I.] Incubation @ 20 °C	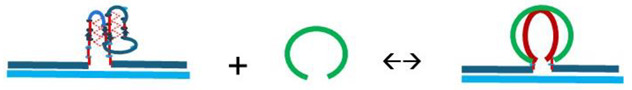	Time
[I.] 2 week Incubation @ 4 °C sample	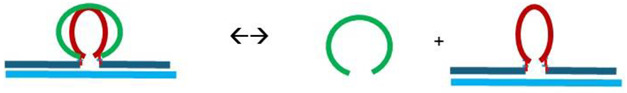	Temperature

**Table 3:** Schematically outlined coupled melting/reannealing processes associated with temperature domain [II.].

Domain [II.] 35 °C < T < 50 °C	Reaction	Comments
[II.]	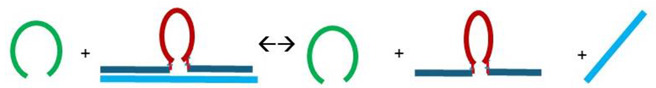	Temperature Coupled reaction
	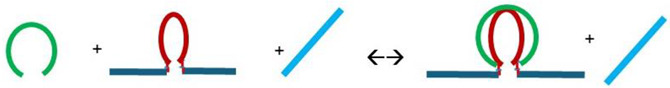	

**Table 4:** Schematically outlined melting/reannealing processes associated with temperature domain [III.].

Domain [III.] 50 °C < T < 70 °C	Reaction	Comments
[III.]	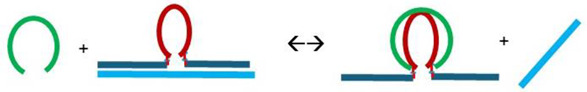	Temperature
	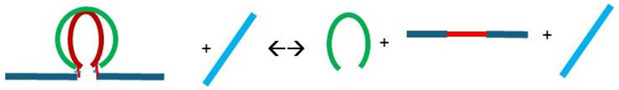	

## Data Availability

Data are contained within the article.
